# Supplementary material for: MYO6 Regulates Spatial Organization of Signaling Endosomes Driving AKT Activation and Actin Dynamics
Source: Cell Rep. 2017 Jun 6;19(10):2088–101. doi: 10.1016/j.celrep.2017.05.048 (PMC5469940; doi:10.1016/j.celrep.2017.05.048)
Supplement: Document S1. Supplemental Experimental Procedures and Figures S1–S7 [file mmc1.pdf]

Cell Reports, Volume *19*

Supplemental Information

MYO6 Regulates Spatial Organization of Signaling Endosomes Driving AKT Activation and Actin Dynamics

Thomas A. Masters, David A. Tumbarello, Margarita V. Chibalina, and Folma Buss

Supplemental Information

Supplemental Experimental Procedures

Cloning of GFP-MYO6+. The 6 IQ domain of MYO5 was inserted into GFP-MYO6-NI (No Insert) by successive rounds of overlap extension PCR. The MYO6 tail was amplified from amino acid 913 to the C-terminal end with 5’GCGTGAGCTGAAGAAACTCAAAttacagaaaaaaaaacagcagg-3’ (the capitalised 5’ end of this oligo corresponds to the end of the 6IQ region of MYO5; amino acid 909) and 5’-TTTgcggccgcTTATTTCAACAGGTTCTGCAGC-3’ (containing a NotI site). The MYO5 motor domain and lever arm (amino acids 1-909) was amplified with oligos 5’-aaagaattctgatggctgcgtcggagctctacaca-3’ and 5’-cctgctgtttttttttctgtaaTTTGAGTTTCTTCAGCTCACGC-3’ (with the capitalised section corresponding to the reverse complement of the 3’ MYO5 sequence). These two products were then combined via an overlap extension reaction.

To create MYO6+, The MYO5 motor domain in this hybrid construct was replaced by the MYO6 motor domain by a similar approach. The motor domain of MYO6 (amino acids 1-770) was amplified by oligos 5’-caagaattcaaatggaggatggaaagccc-3’ and 5’-GGCAGCCCGAAGTTTGTCAGCcatgatctgatcaaattctgc -3’, where the capitalised region corresponds to the reverse complement of the 5’- end of the MYO5 lever arm. The MYO5 lever arm and MYO6-tail fusion were amplified by oligos 5’- gcagaatttgatcagatcatgGCTGACAAACTTCGGGCTGCC-3’ and 5’-TTTgcggccgcTTATTTCAACAGGTTCTGCAGC-3’ (as used in the first round). The two products were then fused by overlap extension to create the final construct; MYO6(1-770):MYO5(763-909):MYO6(913-end). This was cloned into pEGFP-C3 such that GFP was present at the N-terminus once expressed.

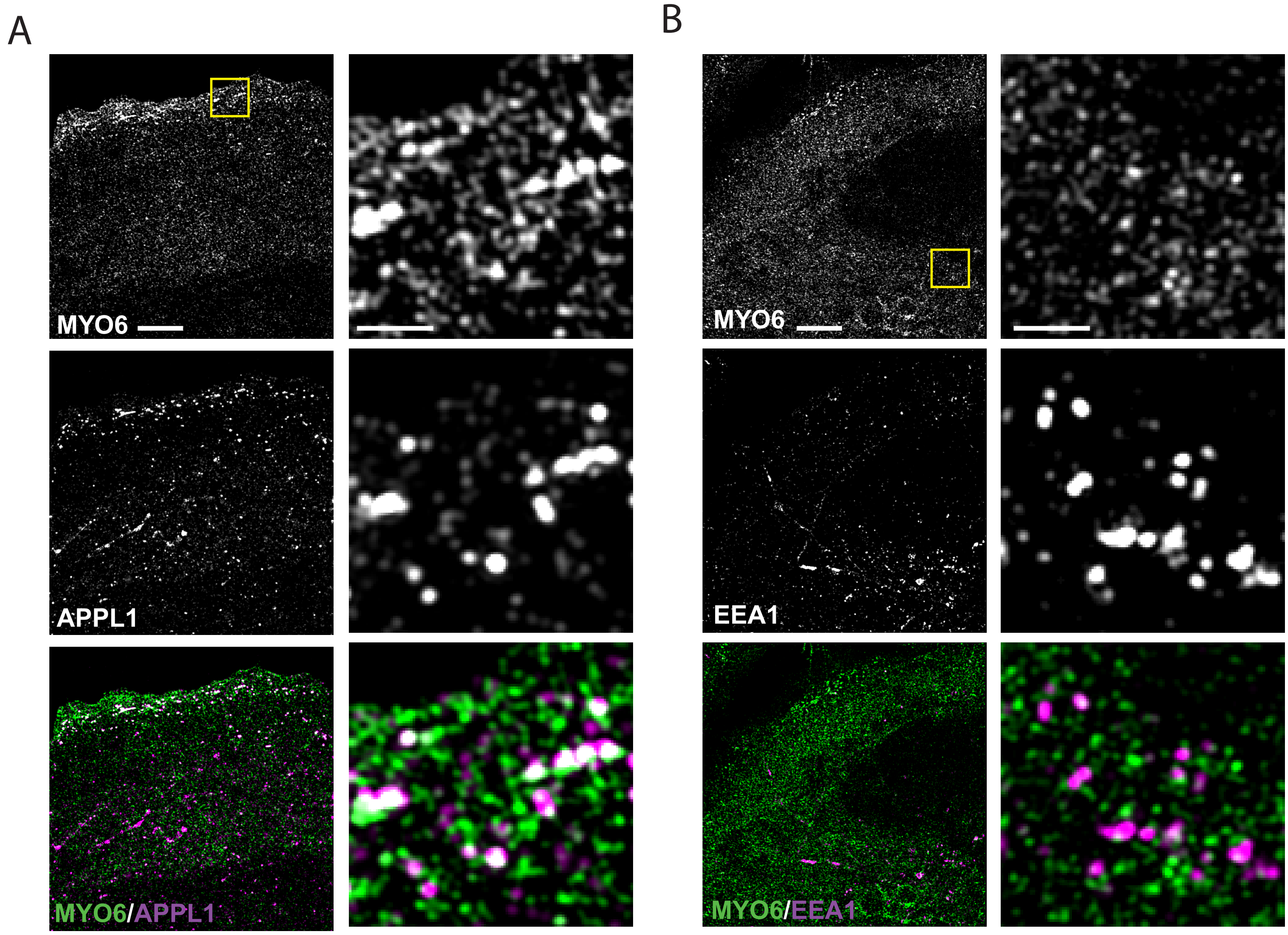

Figure S1, linked to Figure 1. MYO6 localises to APPL1 but not EEA1 endosomes. Hela cells were transfected with GFP-MYO6, fixed and stained with (A) anti-APPL1 polyclonal or (B) anti-EEA1 monoclonal antibodies, followed by imaging with Structured Illumination Microscopy. An overlay of MYO6 with APPL1 (MYO6/APPL1; green and magenta respectively) is presented in (A) together with the respective overlay with EEA1 in (B).

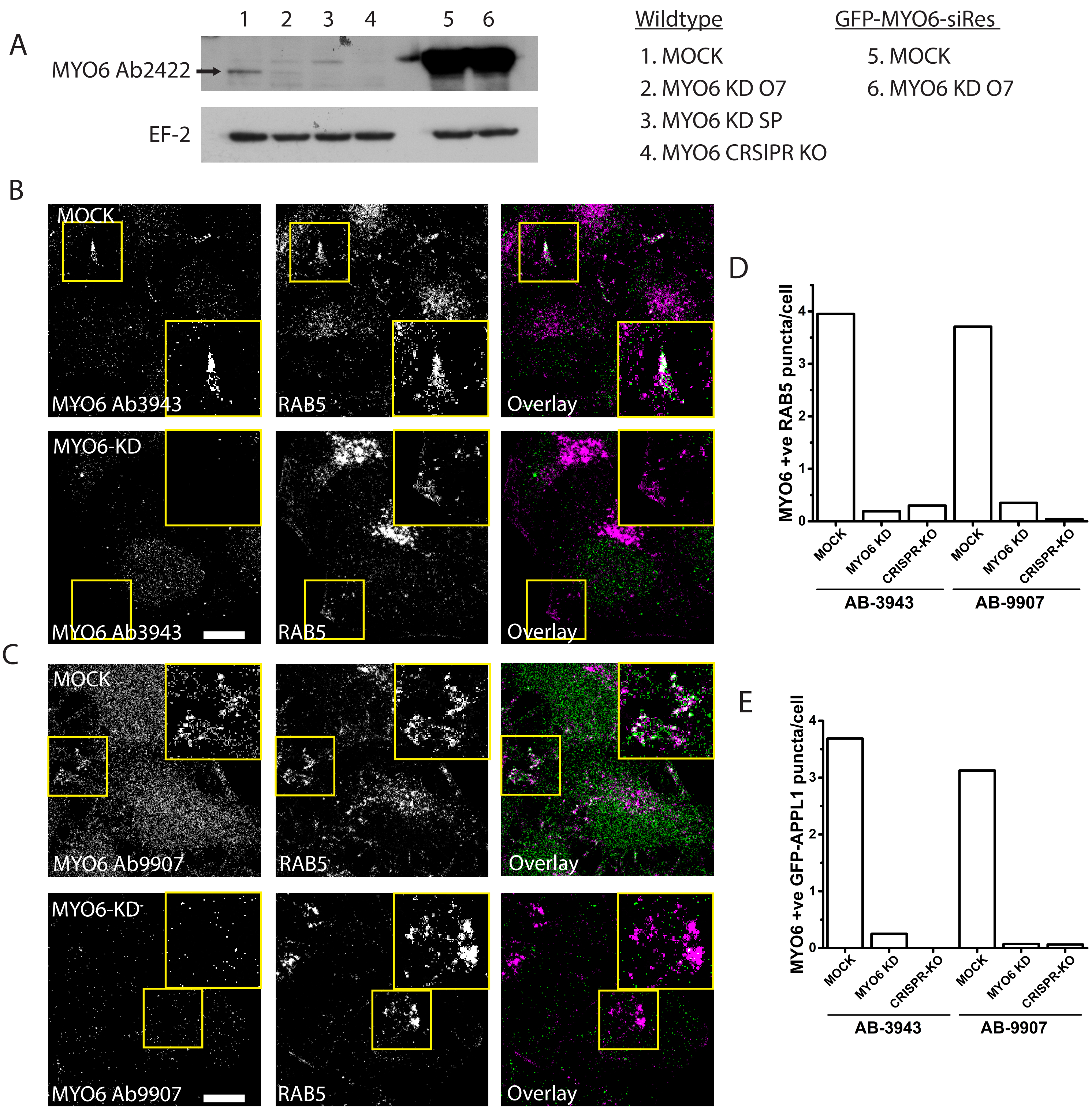

Figure S2, linked to Figure 2. Manipulation of endogenous MYO6 in Hela cells. (A) Western blotting of endogenous MYO6 (with homemade rabbit polyclonal anti-MYO6 antibody 2422) in wildtype and GFP-MYO6-siRes-expressing mock, siRNA Smartpool and O7 treated Hela cells. EF-2 is shown as loading control. (B) A homemade antibody to MYO6 (Ab3943) detects MYO6 on RAB5 puncta in mock but not MYO6 KD cells (scale bar 5 µm). (C) A further homemade antibody to MYO6 (Ab9907) detects MYO6 on RAB5 puncta in mock but not MYO6 KD cells (scale bar 5 µm). (D) Quantification of images in (A) and (B) and MYO6 positive-RAB5 puncta CRISPR MYO6 KO cells. (E) Quantification of GFP-APPL1 puncta positive for MYO6 in Hela MOCK, MYO6-KD and CRISPR MYO6 KO cells.

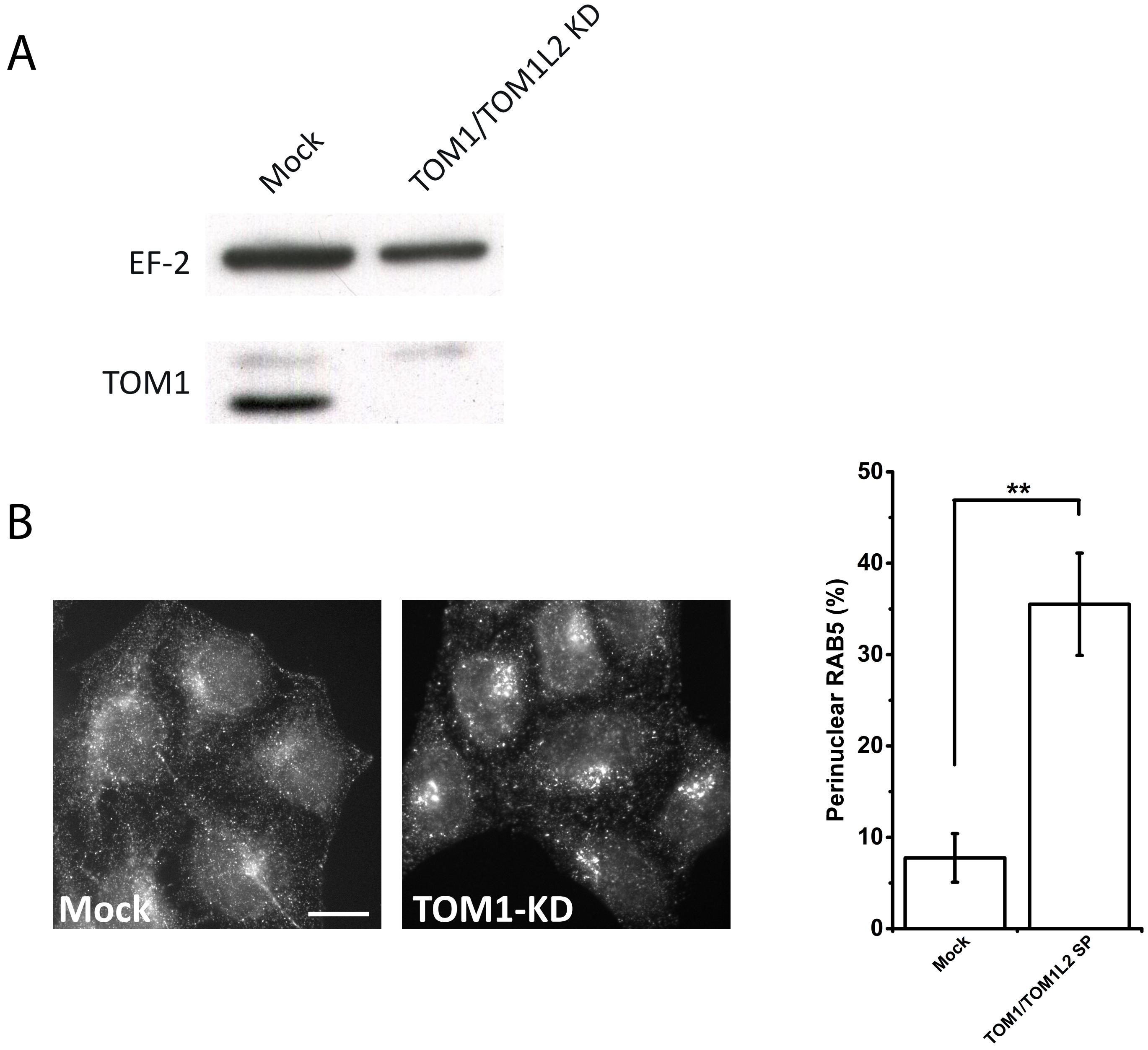

Figure S3, linked to Figure 2. Depletion of the endosomal MYO6 binding partner TOM1 leads to peri-nuclear accumulation of RAB5 endosomes. A) Cells were treated with Mock or siRNA targeting TOM1 and TOM1L2, lysed and subjected to Western blotting for TOM1 (EF-2 was used as a loading control). B) Cells were treated with Mock or siRNA targeting TOM1 and TOM1L2, fixed and stained with anti-RAB5 antibody. Cells with perinuclear accumulation of RAB5 were counted manually. Scale bar 10 µm. At least 300 cells per condition over 3 independent experiments were analysed. Bar graph represent mean ± S.D.

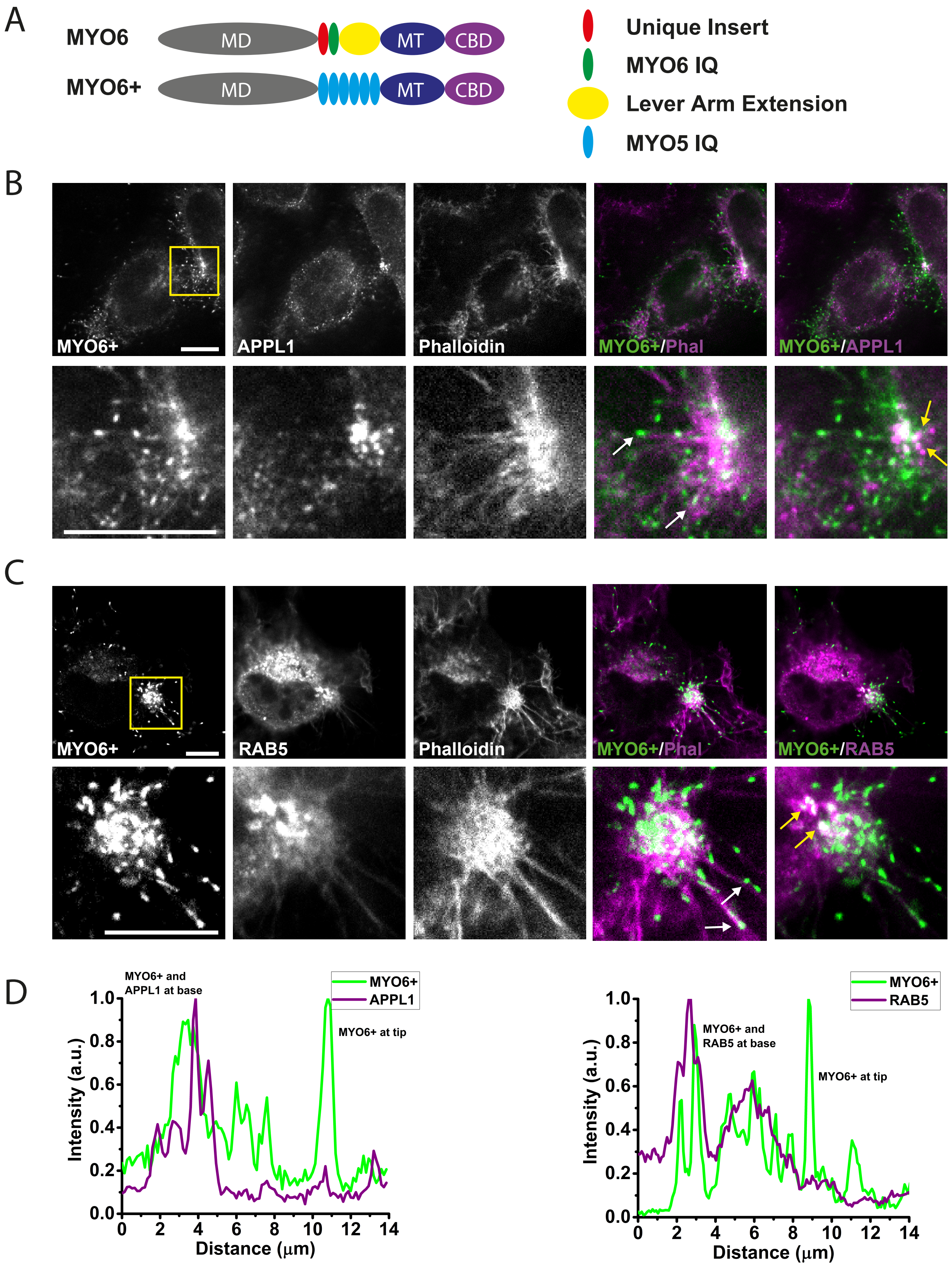

Figure S4, linked to Figure 2. A plus-end directed MYO6 mutant repositions APPL1- and RAB5-positive endosomes and re-organises cortical actin. (A) Schematic layout of MYO6+ and comparison to wild-type MYO6. (B). Hela cells were transfected with GFP-MYO6+, fixed and stained with anti-APPL1 polyclonal antibody and phalloidin. Overlays of mutant MYO6 with phalloidin (green and magenta respectively) and MYO6 with APPL1 (+/AP; green and magenta respectively) are presented. White arrows indicate filopodia tips and yellow arrows indicate APPL1 endosomes at the base. Scale bars 10 µm. (C). Hela cells were transfected with GFP-MYO6+, fixed and stained with anti-RAB5 monoclonal antibody and phalloidin. Overlays of mutant MYO6 with phalloidin (green and magenta respectively) and MYO6 with RAB5 (green and magenta respectively) are presented. White arrows indicate filopodia tips and yellow arrows indicate RAB5 endosomes at the base. Images are representative of three independent experiments. (D) Representative linescans of the images in (B) and (C) showing accumulation of RAB5 and APPL1 with MYO6+ at the base and an additional MYO6+ density at the tips of filopodia. Scale bars 10 µm.

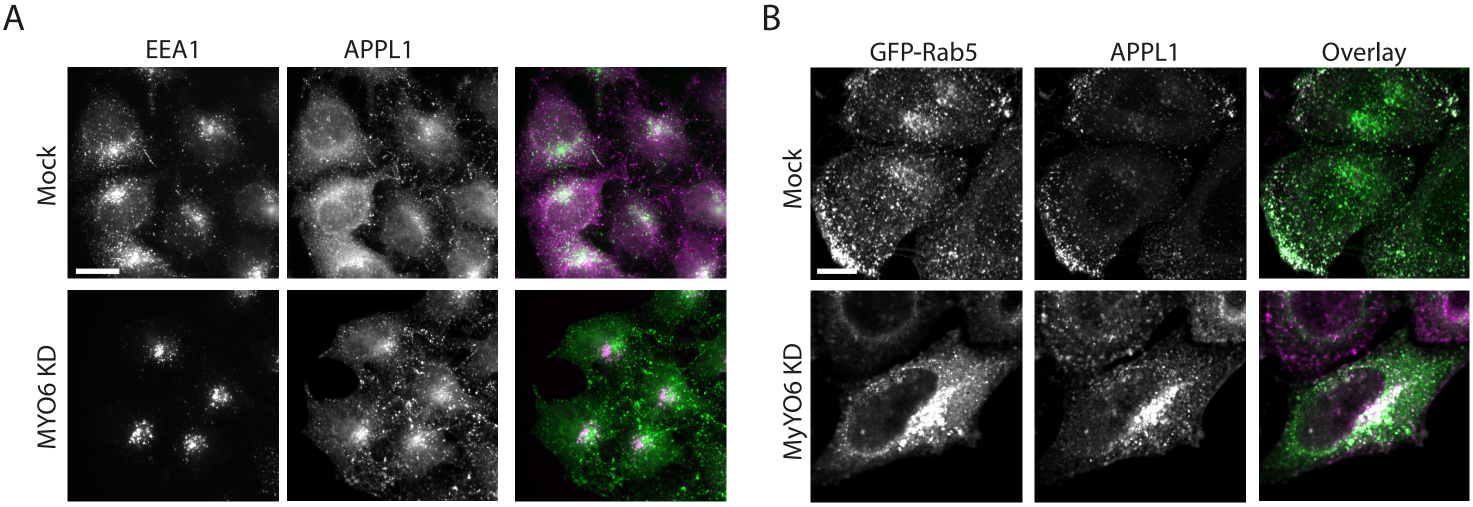

Figure S5, linked to Figure 3. Further characterisation of the endosomal system on acute depletion of MYO6. (A). Cells were treated with siRNA against MYO6, fixed and stained for EEA1 and APPL1. Scale bar 20 µm. (B). Cells were treated with siRNA against MYO6, transfected with GFP-RAB5, fixed and stained for APPL1. Scale bar 10 µm.

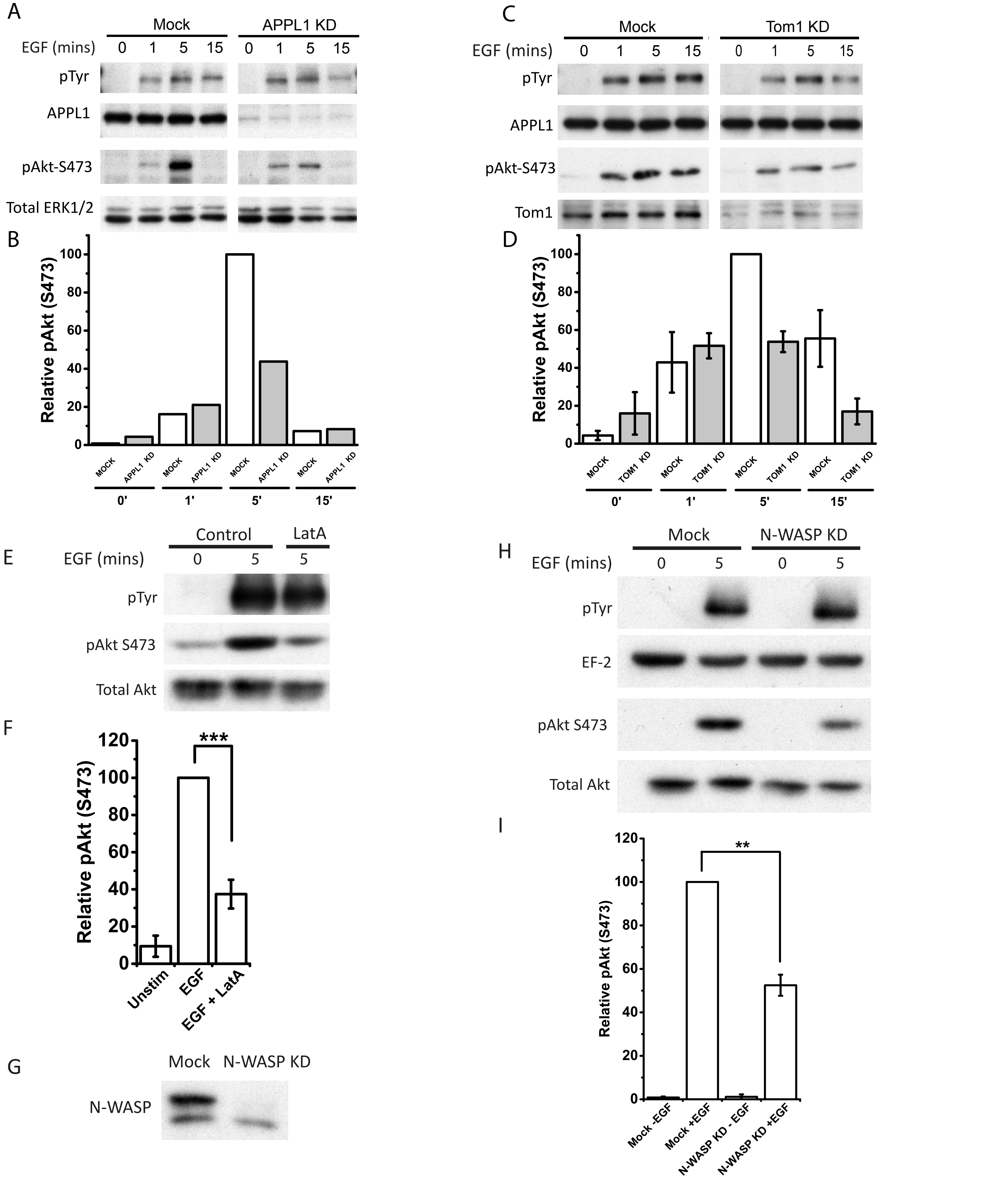

Figure S6, linked to Figure 4. The role of endosomes and actin networks in Akt phosphorylation. (A) A549 cells were treated with MYO6 or APPL1 siRNA and stimulated with 20 ng/ml EGF for the times indicated. Lysates were generated and blotted with the indicated antibodies. (B) Quantification of A. (C) A549 cells were treated with Tom1 siRNA and stimulated with 20 ng/ml EGF for 5 minutes. Lysates were generated and blotted with the indicated antibodies. (D) Quantification of (C) (3 independent experiments). (E) Cells were incubated with LatA, stimulated with EGF and lysates examined by western blot. (F) Quantification of (E) (6 independent experiments). (G) Cells were treated with N-WASP targeting siRNA and lysates were analysed by western blot. (H) Cells were treated with N-WASP targeting siRNA, stimulated with EGF and lysates were analysed by western blot. (I) Quantification of (H) (2 independent experiments). Bar graphs represent mean ± S.E.M.

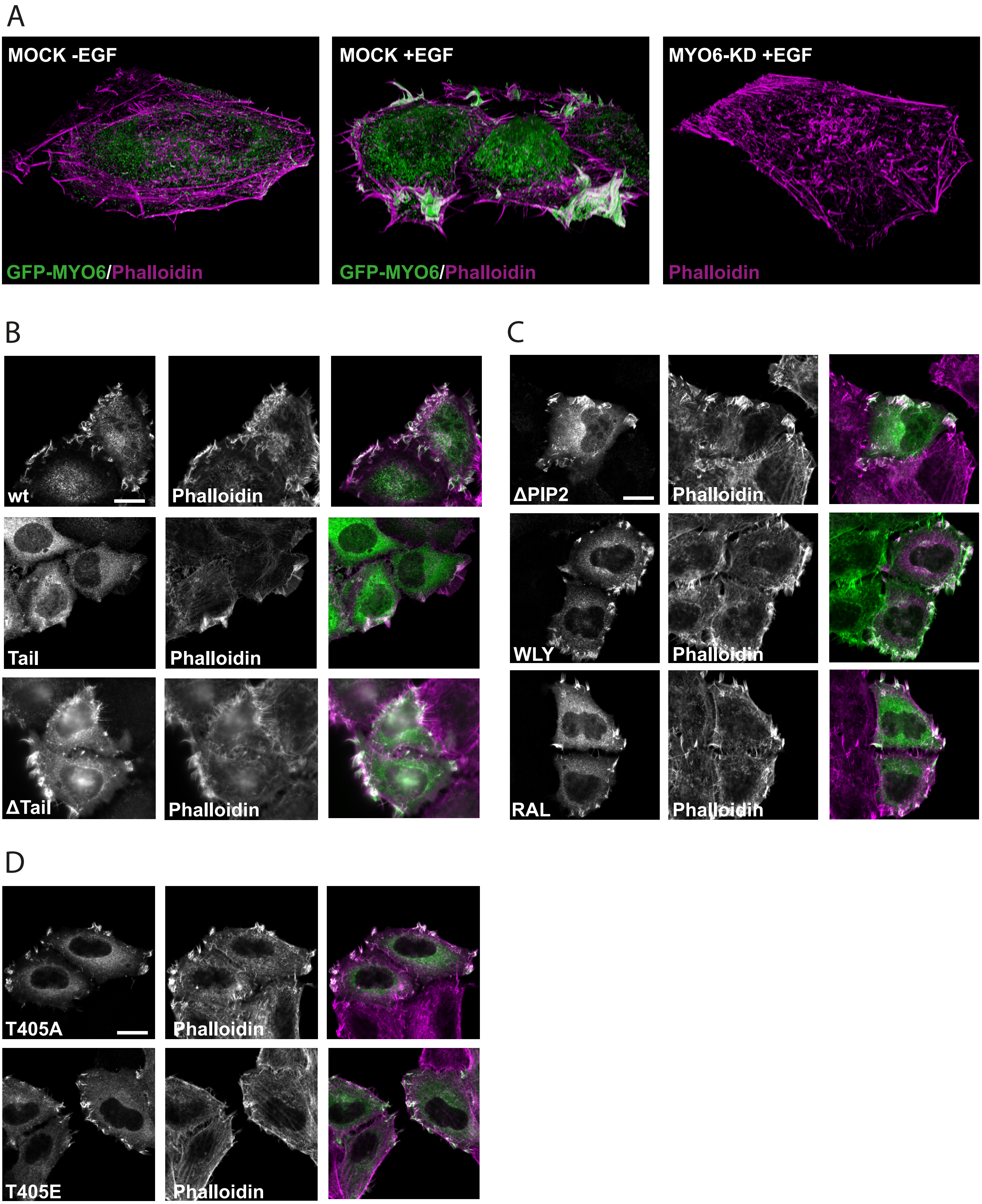

Figure S7, linked to Figure 6. MYO6 localises to ruffles through the head domain, independent of head phosphorylation state. Hela cells were transfected with the indicated MYO6 constructs, stimulated with 100 ng/ml EGF for 5 minutes and fixed. (A) Structured Illumination Microscopy shows both the structure of EGF-induced ruffles containing MYO6 (green), and F-actin structure (labelled by phalloidin, magenta) in mock unstimulated, mock EGF-stimulated and MYO6-siRNA-treated EGF-stimulated cells. (B) Wild-type GFP-MYO6, GFP-MYO6-Tail, GFP-MYO6-ΔTail. (C) GFP-MYO6-ΔPIP2, GFP-MYO6-WLY and GFP-MYO6-RAL. (D) GFP-MYO6-T405A and GFP-MYO6-T405E. Scale bars 20 µm.
