# Supplementary material for: MYO6 Regulates Spatial Organization of Signaling Endosomes Driving AKT Activation and Actin Dynamics
Source: Cell Rep. 2017 Jun 6;19(10):2088–101. doi: 10.1016/j.celrep.2017.05.048 (PMC5469940; doi:10.1016/j.celrep.2017.05.048)
Supplement: Document S2. Article plus Supplemental Information [file mmc2.pdf]

Cell Reports

Article

MYO6 Regulates Spatial Organization of Signaling Endosomes Driving AKT Activation and Actin Dynamics

Graphical Abstract

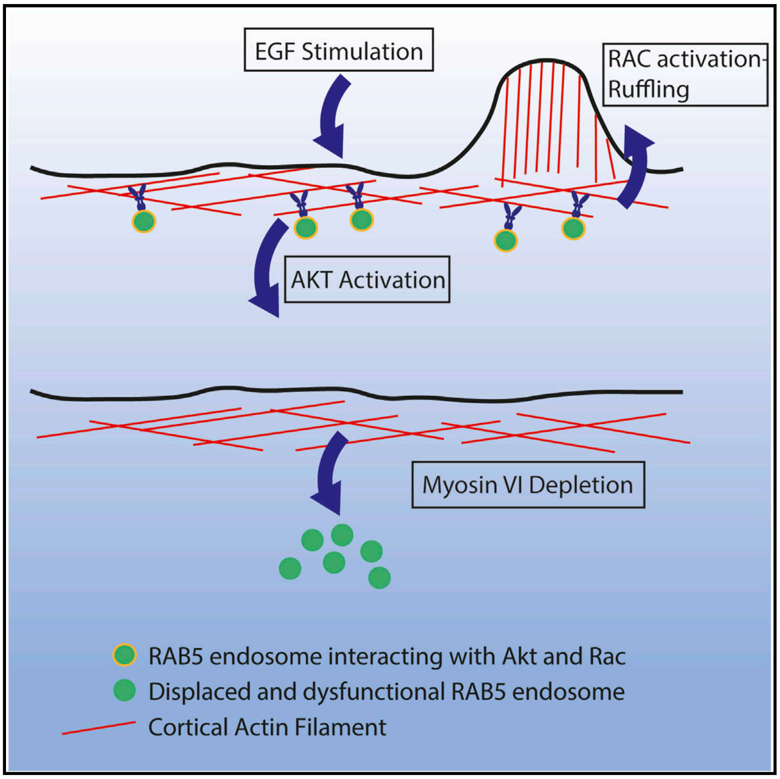

Highlights

- MYO6 tethers APPL1 endosomes to the cell actin cortex
- Loss of MYO6 causes displacement and premature maturation of endosomes
- Perinuclear accumulation of signaling endosomes impairs AKT activation
- MYO6 function is required for cortical actin dynamics

Authors

Thomas A. Masters, David A. Tumbarello, Margarita V. Chibalina, Folma Buss

Correspondence

fb207@cam.ac.uk

In Brief

Masters et al. report that MYO6 tethers APPL1 signaling endosomes to the actin cortex. Depletion of MYO6 leads to premature maturation and displacement of these endosomes away from the cell cortex, as well as defective AKT activation and impaired cortical actin dynamics.

Masters et al., 2017, Cell Reports *19*, 2088–2101
June 6, 2017 © 2017 The Author(s).
http://dx.doi.org/10.1016/j.celrep.2017.05.048

CellPress

OPEN ACCESS
CellPress

Cell Reports
Article

MYO6 Regulates Spatial Organization of Signaling Endosomes Driving AKT Activation and Actin Dynamics

Thomas A. Masters,[1] David A. Tumbarello,[1,2,3] Margarita V. Chibalina,[1,2,4] and Folma Buss[1,5,*]
[1]Cambridge Institute for Medical Research, Wellcome Trust/MRC Building, Hills Road, Cambridge CB2 0XY, UK
[2]These authors contributed equally
[3]Present address: Biological Sciences, Life Sciences Building 85, University of Southampton, Highfield Campus, Southampton SO17 1BJ, UK
[4]Present address: Oxford Centre for Diabetes, Endocrinology and Metabolism, University of Oxford, Churchill Hospital, Headington, Oxford OX3 7LE, UK
[5]Lead Contact
*Correspondence: fb207@cam.ac.uk
http://dx.doi.org/10.1016/j.celrep.2017.05.048

SUMMARY

APPL1- and RAB5-positive signaling endosomes play a crucial role in the activation of AKT in response to extracellular stimuli. Myosin VI (MYO6) and two of its cargo adaptor proteins, GIPC and TOM1/TOM1L2, localize to these peripheral endosomes and mediate endosome association with cortical actin filaments. Loss of MYO6 leads to the displacement of these endosomes from the cell cortex and accumulation in the perinuclear space. Depletion of this myosin not only affects endosome positioning, but also induces actin and lipid remodeling consistent with endosome maturation, including accumulation of F-actin and the endosomal lipid PI(3)P. These processes acutely perturb endosome function, as both AKT phosphorylation and RAC-dependent membrane ruffling were markedly reduced by depletion of either APPL1 or MYO6. These results place MYO6 and its binding partners at a central nexus in cellular signaling linking actin dynamics at the cell surface and endosomal signaling in the cell cortex.

INTRODUCTION

The spatial distribution of cell signaling is a crucial aspect of cell regulation and the sensing of extracellular environmental cues. In particular, polarized cellular functions such as migration rely on spatial regulation of RAC activation and membrane trafficking at the leading edge. The mechanisms underlying cell polarity and directed cell migration have been extensively investigated, but many questions still remain (Petrie et al., 2009). In particular, spatial heterogeneity and structure in the endosomal system is emerging as a key regulator of signaling processes (Sadowski et al., 2009). For instance, the positioning of lysosomes is mediated by motors linked to the microtubule cytoskeleton and has long been shown to be important for their cellular function (Heuser, 1989; Korolchuk et al., 2011). The distribution of many other classes of organelles is also correlated with the actin and microtubule cytoskeletons, with interactions mediated by actin- and tubulin-based molecular motors. In the case of signaling endosomes, which are anchored to cortical actin near the cell surface, this may enable the integration of external geometric or mechanical cues with the endosomal system, although the underlying mechanisms have not been determined. Thus, understanding how endosomes interact with the actin cytoskeleton and the consequences for their function is of crucial importance.

The role of actin dynamics at several steps along the endocytic pathway has been given increasing attention. At the plasma membrane, coated pits require actin polymerization to complete scission when membrane tension is high, such as at the apical surface of polarized cells (Boulant et al., 2011). At early sorting endosomes, WASH-dependent actin polymerization regulates the cargo sorting required for recycling and transport of receptors to lysosomes (Duleh and Welch, 2010). However, the role of the cortical actin network underneath the plasma membrane in positioning and function of early signaling endosomes, which connect clathrin-coated vesicles and EEA1-positive sorting endosomes, is much less understood.

AKT (or Protein Kinase B) is a widely expressed serine/threonine kinase with crucial roles in the transduction of mitogenic signals to the nucleus (Manning and Cantley, 2007) and in cell migration and metastasis (Broussard et al., 2012; Irie et al., 2005; Kim et al., 2001). Downstream signaling from AKT determines decision making during cell growth, division, and survival. AKT is a major target of the PI-3 Kinase (PI3K) pathway and is frequently hyper-activated in a range of cancers, receiving much attention as a potential therapeutic target (Cheng et al., 2005). The mechanism of AKT activation has been extensively studied. In the canonical model, binding of extracellular growth factors to receptor tyrosine kinases induces their activation via conformation change and trans-phosphorylation (Lemmon and Schlessinger, 2010). Phosphorylated receptors recruit adaptor proteins such as GRB2 and SOS, which, in turn, recruit Ras and PI3K to the plasma membrane. Activated PI3K generates the phospholipid PI(3,4,5)P_3, which is recognized and bound by the PH domain of cytosolic AKT. This triggers a conformational change in AKT (Calleja et al., 2007), which is coupled to

 © 2017 The Author(s).
This is an open access article under the CC BY license (http://creativecommons.org/licenses/by/4.0/).

phosphorylation at T308 by PDK1 and at the S473 site by the mTORC2 complex (Ikenoue et al., 2008; Sarbassov et al., 2005).

However, AKT activation due to growth factor signaling not only depends on interactions at the plasma membrane but can also occur on endosomes (Wang et al., 2002). In particular, signaling endosomes characterized by the presence of APPL1 (adaptor protein, phosphotyrosine interacting with PH domain, and leucine zipper 1) have been shown to be required for AKT activation in cultured cells and zebrafish (Schenck et al., 2008). APPL1 binds directly to AKT (Mitsuuchi et al., 1999) and is required for AKT activation in response to several extracellular stimuli including insulin (Saito et al., 2007), nerve growth factor (Lin et al., 2006; Varsano et al., 2006), and lysophosphatidic acid (LPA) (Varsano et al., 2012).

MYO6 is an actin-based motor, which functions in a wide range of important cellular processes, including endocytosis (Buss et al., 2001), polarized secretion (Chibalina et al., 2010), and autophagy (Tumbarello et al., 2012). It is unique in that it is the only myosin known to move toward the minus end of actin filaments (Wells et al., 1999). We have previously shown that MYO6 localizes to EGF-stimulated membrane ruffles (Buss et al., 1998) and is required for the formation of ruffling protrusions during wound healing and directed cell migration (Chibalina et al., 2010). Furthermore, MYO6 is upregulated in numerous cancers, where it has been suggested to promote cell migration and invasion (Dunn et al., 2006) and thus represents a potential therapeutic target. MYO6 has several splice isoforms, of which the Large Insert isoform is preferentially expressed in polarized epithelia and functions in clathrin-mediated endocytosis at the apical surface (Buss et al., 2001). In this study, we focus on the No Insert isoform of MYO6, which has been reported to localize to signaling endosomes through its adaptor proteins GIPC and TOM1/TOM1L2 (Tumbarello et al., 2012), although the function of MYO6 and its binding partners on these endosomes is not known.

In this study, we performed a detailed analysis on the role of MYO6 at this specific class of signaling endosomes. We demonstrate that MYO6 not only localizes to cortical APPL1 endosomes, but loss of MYO6 leads to perturbation of both their localization and composition, which, in turn, impairs their function in AKT activation. MYO6 depletion either by small interfering RNA (siRNA) knockdown or in knockout mouse embryonic fibroblasts (MEFs) or depletion of its binding partner TOM1 leads to a reduction of AKT phosphorylation in response to EGF stimulation. MYO6 is also present at high levels in EGF-stimulated membrane ruffles and is required for their formation, suggesting crucial links between actin cytoskeleton dynamics at the plasma membrane and endosomal signaling in the cell cortex.

RESULTS

MYO6-Positive APPL1 Endosomes Align along Cortical Actin Filaments

We have previously reported that MYO6 and its cargo adaptor proteins GIPC and TOM1/TOM1L2 are present on RAB5 and APPL1-positive signaling endosomes (Tumbarello et al., 2012). These endosomes are predominantly located in the actin-rich cell cortex. To examine whether the distribution of APPL1 endosomes is linked to actin filaments, we used structured illumination microscopy (SIM) to visualize APPL1 endosomes and cortical actin filaments with high resolution in fixed HeLa cells (Figure 1A). Our SIM images demonstrate that the vast majority of APPL1 endosomes are positive for MYO6 (Figure S1A) and align along actin filament bundles (Figure 1A). This arrangement is in contrast to that of EEA1-positive endosomes, which are surrounded by actin filament patches (Figure 1B) and do not colocalize with MYO6 (Figure S1B). These results indicate that, while both APPL1- and EEA1-positive endosomes are associated with actin, the architecture and geometry of these interactions are highly divergent.

Localization of APPL1 Endosomes to the Actin Cortex Depends on MYO6

To assess the role of MYO6 in linking these signaling endosomes to actin filaments, we treated cells with MYO6-targeting siRNA (Figure 2A). Depletion of MYO6 by a smartpool of siRNA oligos led to a dramatic accumulation of the RAB5- and APPL1-positive endosomes in the perinuclear space (Figures 2A and 2B). Depletion of endogenous MYO6 from endosomes was confirmed by western blotting and immunostaining with two rabbit polyclonal antibodies (Figures S2A–S2E). We verified this result using a single oligo targeting MYO6 (O7) and by phenotypic rescue following stable expression of the corresponding siRNA-resistant GFP-MYO6 (Figures 2C and 2D). Accumulation of RAB5 endosomes was also observed on depletion of the endosomal MYO6-binding partner TOM1 (Figures S3A and S3B). Perinuclear accumulation of RAB5 endosomes was accompanied by a visible accumulation of F-actin (Figure 2E) and a slight reduction of associated APPL1 (Figure 2A). These results suggest that MYO6 promotes tethering of signaling endosomes to cortical actin, effectively opposing long-range movement on microtubules (Flores-Rodriguez et al., 2011) and associated endosomal maturation (Driskell et al., 2007). To test this hypothesis, we depleted MYO6 and incubated cells with the microtubule depolymerizing agent nocodazole (Figures 2E and 2F). While not rescuing peripheral endosome localization, nocodazole prevented accumulation of endosomes in the perinuclear space (Figures 2E and 2F), indicating a role for MYO6 in tethering peripheral signaling endosomes.

Mutant Plus-End-Directed MYO6 Causes Clustering of APPL1 Endosomes in the Cell Cortex

To further test the role of MYO6 in endosome positioning, we engineered a mechanical mutant of MYO6, which moves toward the plus end of actin filaments (Masters and Buss, 2017). Replacement of the MYO6 lever arm with the lever arm of the plus-end-directed motor myosin V (MYO5) has previously been shown to direct MYO6 movement toward the plus end of actin filaments in vitro (Park et al., 2007). In our recent study, we likewise replaced the lever arm (including the unique insert, IQ, and lever arm extension) with the 6IQ domains from MYO5 but retain the motor domain and the cargo-binding tail of MYO6 to preserve interaction with endogenous cargoes (Figure S4A). This construct (termed MYO6^{+}) still targets to APPL1 endosomes but translocates these endosomes to the base of actin-rich

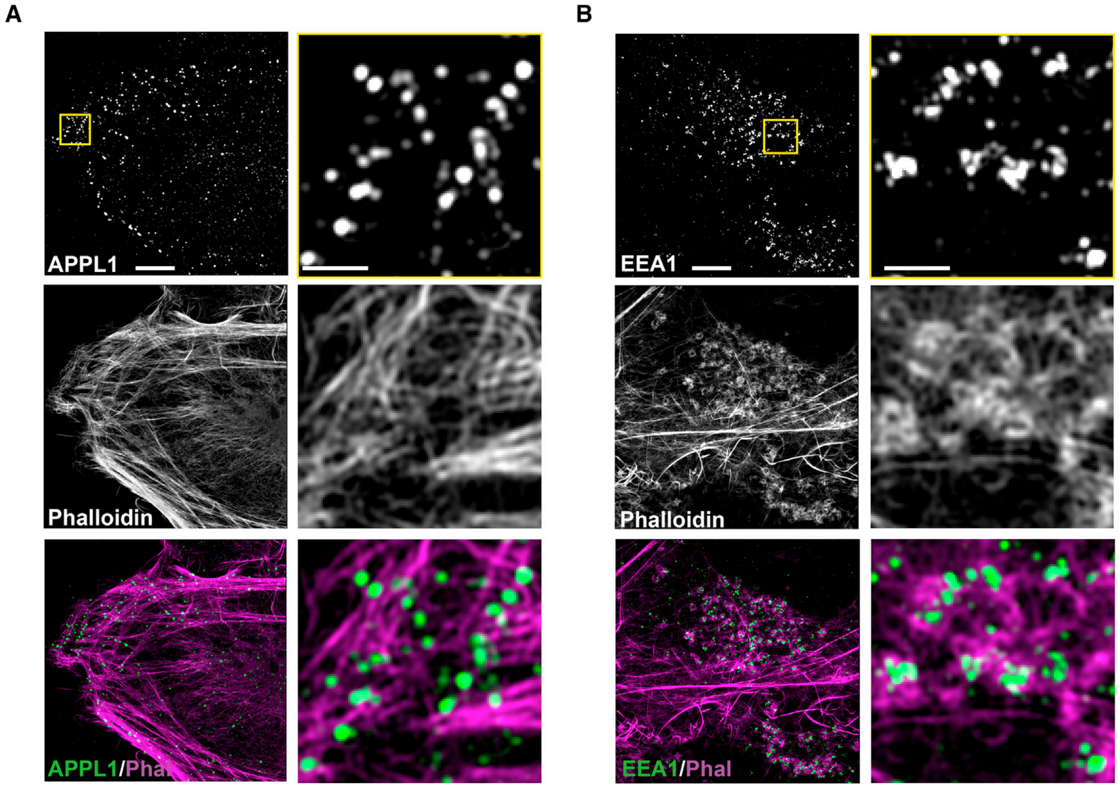

Figure 1. SIM of Endosomes and the Local Actin Network

HeLa cells were fixed and stained with (A) anti-APPL1 polyclonal or (B) anti-EEA1 monoclonal (detected with Alexa-647-labeled secondary antibodies), and phalloidin conjugated with Alexa 568. Overlays of MYO6 and APPL1 (MYO6/APPL1; green and magenta, respectively) and APPL1 with phalloidin (APPL1/Phal; magenta and cyan, respectively) are presented in (A) together with the respective overlays for EEA1 in (B). Scale bars in the first column, 5 μm, and in the second column, 1 μm.

protrusions at the plasma membrane resembling filopodia (Figure S4B). GFP-MYO6^{+} accumulated at both the tips of filopodia (indicating that this mutant MYO6 indeed functions as a plus-end-directed motor in cells) and on the APPL1 endosomes jammed and clustered at the cell surface underneath the filopodia. Staining for RAB5 confirmed a similar accumulation of endosomes (Figures S4C and S4D). These results support our finding that MYO6 plays a crucial role in positioning of signaling endosomes.

Loss of MYO6 Leads to Accelerated Maturation of APPL1 Endosomes

We next determined whether aberrant endosome localization and positioning in MYO6-depleted cells causes any changes in the biochemical composition of these vesicles. The forward trafficking in the endocytic pathway and maturation of APPL1 endosomes into EEA1 endosomes is marked by the accumulation of PI(3)P (Zoncu et al., 2009). We therefore first transfected cells with the PI(3)P biosensor GFP-PX, which revealed a high density of PI(3)P (Figure 3A) and EEA1 (Figure S5A) present on the endosomes that accumulated in the perinuclear space. This indicates that the redistribution of signaling endosomes toward the cell center, caused by loss of MYO6, leads to a maturation process including accumulation of PI(3)P, potentially promoting detachment of APPL1 (consistent with the observed slightly weaker perinuclear accumulation of APPL1 relative to RAB5). Expression of GFP-RAB5 leads to enhanced retention of APPL1 on the displaced endosomes (Figure S5B), as previously suggested (Zoncu et al., 2009). Loss of MYO6 is associated with an accumulation of both PI(3)P (Figures 3B and 3C) and F-actin (Figure 3D) in the perinuclear compartment. These experiments show that MYO6 plays an important role in the spatiotemporal distribution of signaling endosomes and their biochemical characteristics.

AKT Activation Is Reduced in MYO6-Depleted Cells

The activation of the Ser/Thr kinase AKT downstream of growth factor stimulation not only has been shown to take place at the plasma membrane, but it continues after receptor endocytosis on APPL1 signaling endosomes (Schenck et al., 2008). We thus determined whether cellular responses to EGF stimulation were perturbed by aberrant signaling endosome positioning after depletion of MYO6. Our results demonstrate that knockdown of MYO6 in A549 cells significantly reduced AKT activation following EGF stimulation, as determined by phosphorylation of S473 but not T308 (Figures 4A and 4B). A similar reduction in phosphorylated AKT (pAKT) was observed in primary mouse fibroblasts derived from the MYO6 knockout mice (Snell's

OPEN ACCESS
CellPress

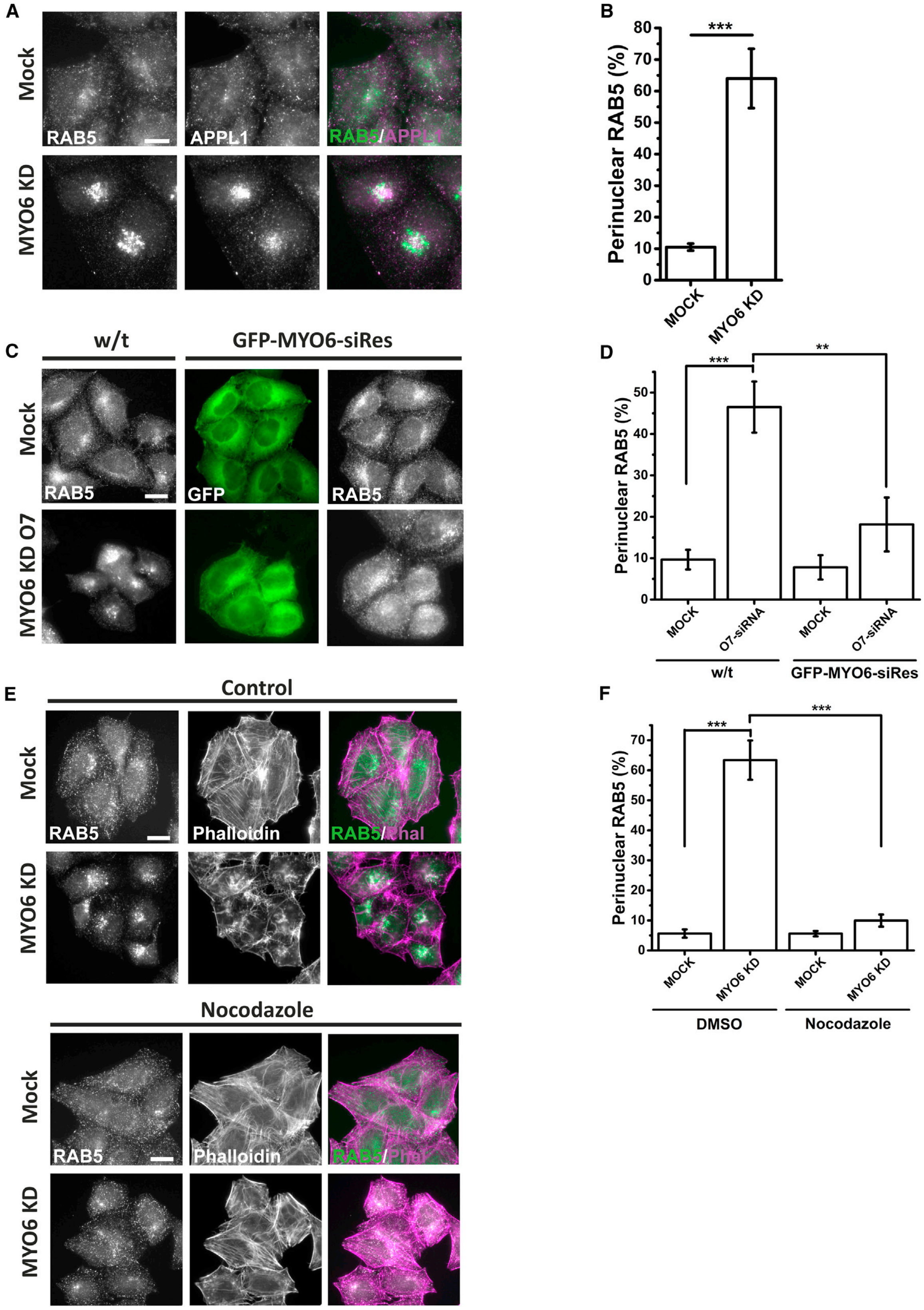

(legend on next page)

OPEN
ACCESS
CellPress

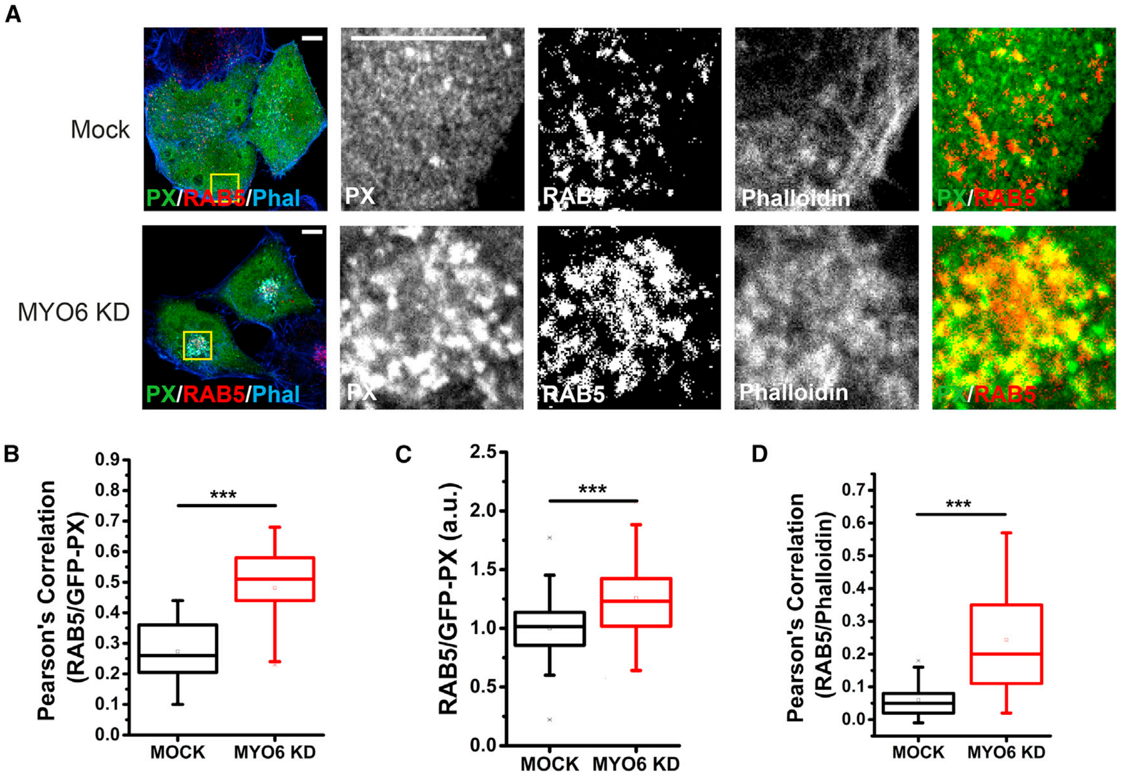

Figure 3. Loss of MYO6 Leads to Lipid and Actin Remodeling of RAB5 Endosomes

(A) HeLa cells were treated with MYO6 targeting siRNA, transfected with GFP-PX, fixed, and stained with anti-RAB5 monoclonal antibody and phalloidin. An overlay of PX, RAB5, and phalloidin (green, red, and blue, respectively) is presented, with the expanded region showing PX and RAB5 only. Scale bars, 10 μm.

(B) Box-and-whisker plot of Pearson's correlation coefficients for colocalization of RAB5 with GFP-PX in experiments shown in (A).

(C) Box-and-whisker plot of relative enrichment of GFP-PX on RAB5 endosomes (calculated as described in the Experimental Procedures). The mean enrichment in MYO6 KD cells is 1.26 (i.e., a 26% increase in density of PI3P).

(D) Box-and-whisker plot of Pearson's correlation coefficients for colocalization of RAB5 with phalloidin in experiments shown in (A). Each distribution (in B–D) contains over 60 cells from three experiments ($p < 0.001$). Scale bars, 10 μm.

Waltzer - sv) (Figure 4C). Confirming the role of signaling endosomes in AKT activation, depletion of APPL1 (Figures S6A and S6B) resulted in a marked reduction of AKT activation, as reported previously (Schenck et al., 2008). MYO6 is recruited to signaling endosomes via a number of binding partners including TOM1 (Tumbarello et al., 2012). Consistent with this, depletion of TOM1 also significantly reduced AKT phosphorylation following EGF stimulation (Figures S6C and S6D). To analyze further the role of the actin cytoskeleton and actin-based motors in AKT activation, we depolymerized F-actin using latrunculin (LatA) prior to EGF stimulation (Figures S6E and S6F). This led to a significant reduction in phosphorylated AKT, as previously seen in response to insulin (Eyster et al., 2005). However, actin is likely to be required at many stages in EGF stimulation, for instance, the local organization of receptors (Stabley et al., 2013), and indeed less phosphorylated receptor was detected under LatA treatment. To perturb the actin cytoskeleton in a more specific way, we treated cells with N-WASP targeting siRNA (Figure S6G), which also caused a significant reduction in S473 pAKT, with little effect on receptor phosphorylation (Figures S6H and S6I). A similar result was obtained when treating with the N-WASP inhibitor Wiskostatin (data not shown). In summary, these data

Figure 2. MYO6 Is Required for Peripheral Localization of RAB5- and APPL1-Positive Signaling Endosomes

(A) HeLa cells were treated with MYO6 targeting siRNA, fixed and stained with anti-RAB5 monoclonal and anti-APPL1 polyclonal antibodies, and imaged by wide-field microscopy. Scale bar, 10 μm.

(B) Quantification of experiments shown in (A) (more than 500 cells per condition over three independent experiments, $p < 0.001$).

(C) HeLa cells either wild-type or stably expressing siRNA-resistant GFP-MYO6 were treated with a single oligo (O7) targeting MYO6.

(D) Quantification of experiments shown in (C) (more than 600 cells per condition over three independent experiments, $p < 0.001$, **$p < 0.01$).

(E) HeLa cells were treated with MYO6 targeting siRNA, incubated with or without 15 μM nocodazole for 1 hr, fixed, and stained with anti-RAB5 monoclonal and Alexa-568-conjugated phalloidin.

(F) Quantification of experiments shown in (E) (more than 400 cells per condition over three independent experiments, $p < 0.001$).

Images are representative of three independent experiments. Bar graphs represent mean ± SD. Scale bars, 20 μm.

OPEN ACCESS
CellPress

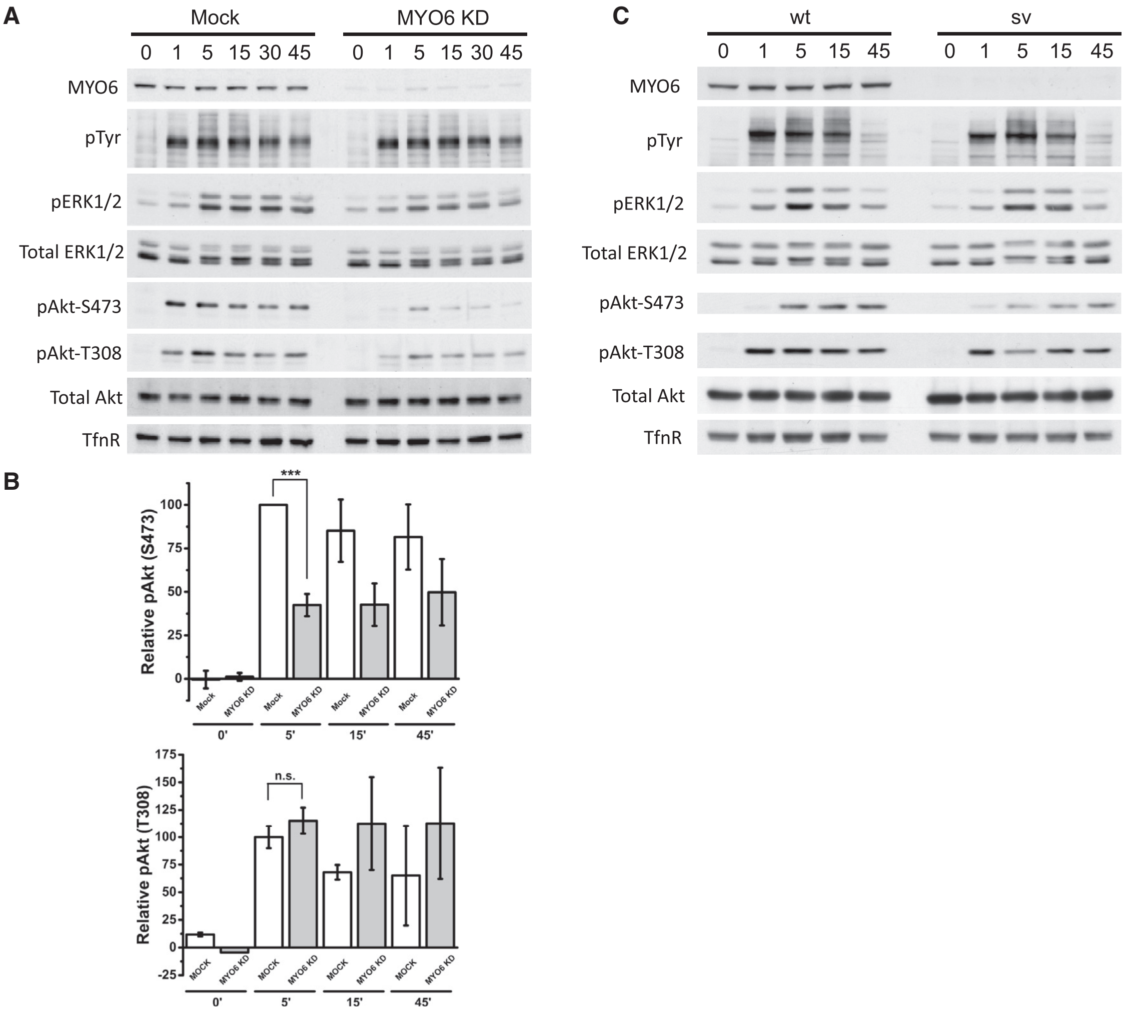

Figure 4. MYO6 Is Required for Full AKT Activation

(A) A549 cells were treated with control or MYO6 siRNA and stimulated with 20 ng/mL EGF for the indicated time periods. Lysates were generated and blotted with the indicated antibodies. pTyr indicates a phosphorylated band at 250 kDa (corresponding to EGFR).

(B) Quantitation of A549 cell lysate blots for pT308 and pS473, showing an average of four independent experiments (MOCK cell lysates in white, MYO6 KD in gray, errors SEM).

(C) Snell's Waltzer fibroblasts (sv) were stimulated with platelet-derived growth factor (PDGF) and lysates examined by western blot.

indicate that F-actin assembly and MYO6-motor functions are crucial in regulating AKT activation.

MYO6 Is Not Required for PI3K Activation and AKT Recruitment to the Plasma Membrane

To assess whether the reduction of AKT activation was caused by processes occurring at the plasma membrane or on signaling endosomes, cells were transfected with GFP-p85α, the regulatory component of the PI3K complex. In unstimulated cells p85α localized to focal adhesions (Figure 5A) (Chen and Guan, 1994), however, upon EGF stimulation, GFP-p85α translocated from focal adhesions to puncta at or close to the plasma membrane (Figure 5A) (Gillham et al., 1999). These puncta colocalized with EGFR (Figures 5A and 5B), indicating that p85α was being recruited into activated clusters of EGFR. No difference in the recruitment of p85α into these clusters was observed upon knockdown of MYO6, strongly suggesting that MYO6 is not required for recruitment and activation of the PI3K complex on the plasma membrane.

Although our results demonstrate that p85α recruitment is not regulated by MYO6, we next determined whether PI3K activity at the plasma membrane was affected by loss of MYO6. For this purpose, we designed a total internal reflection fluorescence (TIRF) assay in which we transfected cells with the GFP-AKT-PH domain, which binds to the 3′-phosphoinositide products of PI3K, PI(3,4)P_2, and PI(3,4,5)P_3. To account for the detectable membrane area at the surface in the TIRF field, we used a fluorescently tagged control plasmid targeted to the plasma membrane, e.g., either PMT-RFP or Lyn-mCherry-FRB. Live cells were imaged in TIRF mode (Figure 5C), and, on addition

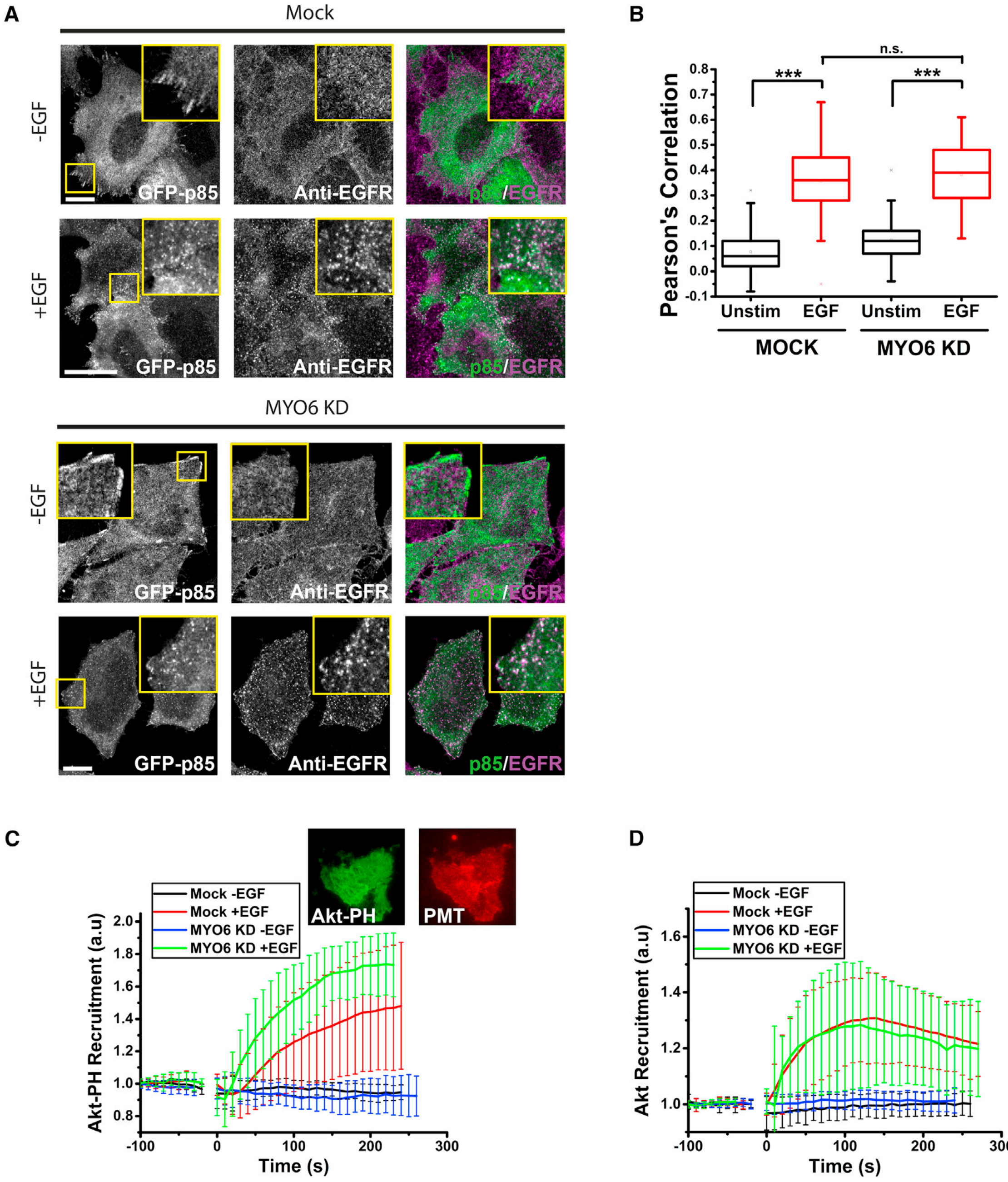

Figure 5. Loss of MYO6 Does Not Affect PI3K Activation or AKT Recruitment to the Plasma Membrane

(A) Cells were transfected with GFP-p85, fixed, and stained with anti-EGFR antibody. Images were acquired by confocal microscopy. Scale bars, 10 μm.

(B) Box-and-whisker plot of Pearson's correlation coefficients for colocalization of GFP-p85 with EGFR in experiments shown in (A) (each distribution contains 60 cells from three independent experiments).

(C) Cells were transfected with GFP-AKT-PH, starved, and imaged by live-cell TIRF microscopy during addition of EGF (data shown are averages of more than 18 cells per condition from three independent experiments).

(D) Cells were transfected with GFP-AKT, starved, and imaged by live-cell TIRF microscopy during addition of EGF (data shown are averages of more than 15 cells per condition from three independent experiments).

Error bars shown are SD.

of EGF, a rapid and sustained recruitment of GFP-AKT-PH to the membrane was observed over 4 min. On average, no significant difference in recruitment was observed between mock and MYO6 knockdown cells, indicating no change in the production of 3′-phosphoinositides by PI3K. To complement this approach, we repeated the experiment using GFP-tagged full-length AKT

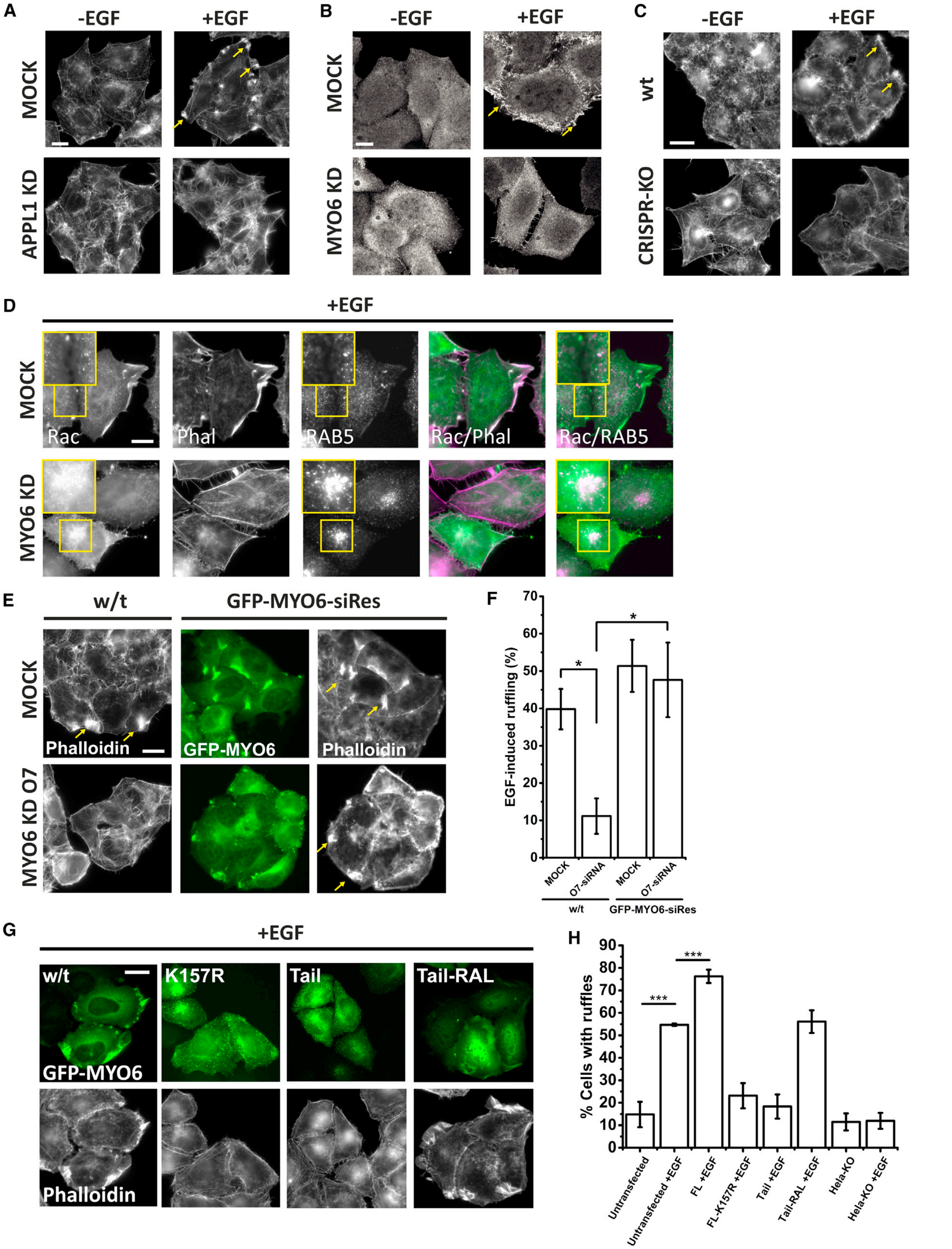

(legend on next page)

(Figure 5D). In this case, a peak in AKT fluorescence intensity indicating maximal AKT recruitment to the plasma membrane was observed after approximately 2 min. Similar to the AKT-PH domain, no difference in recruitment dynamics of full-length AKT was detectable, confirming that plasma membrane AKT activation is unaffected by loss of MYO6.

MYO6 Is Required for Membrane Ruffling

Early RAB5-positive endosomes are important signaling platforms for the activation of RAC, which is locally recycled back to the plasma membrane initiating a spatially restricted reorganization of the actin cortex and formation of plasma membrane protrusions required for cell migration (Palamidessi et al., 2008). As MYO6 is not only present in EGF-stimulated ruffles (Buss et al., 1998) but is also required for directed cell migration (Chibalina et al., 2010), it is conceivable that MYO6 may play a role in endosomal RAC activation and/or trafficking and capture of active RAC within ruffles. We thus next analyzed whether the spatial and biochemical perturbations of peripheral RAB5 (i.e., APPL1)-positive signaling endosomes in MYO6-depleted cells has an impact on RAC activation at the plasma membrane. Consistent with this function, ablation of signaling endosomes by siRNA-mediated knockdown of APPL1 prevented formation of membrane ruffles following stimulation with EGF (Figure 6A). We next analyzed the requirement of MYO6 for ruffle formation. Downregulation of MYO6 by either siRNA (Figures 6B and S7A) or CRISPR-mediated KO (Figure 6C) significantly perturbed ruffle formation following EGF treatment. To understand how RAC activity was affected, we transfected cells with YFP-RAC (Figure 6D). We found that, in wild-type cells, RAC localized in membrane ruffles as expected. When MYO6 was depleted, RAC was no longer localized with peripheral actin (Figure 6D). The defect in membrane ruffling was replicated by a treatment with a single oligo (O7) and was rescued by stable expression of siRNA-resistant GFP-MYO6 (Figures 6E and 6F). Overexpression of either the MYO6 tail or a rigor mutant of MYO6 (K157R), which constitutively binds tightly to actin, prevented ruffle formation, while overexpression of wild-type MYO6 led to an increase in EGF-stimulated membrane ruffling following starvation (Figures 6G and 6H). Conversely, expression of a mutant tail (RAL), which is unable to bind to endosomes, has no effect on ruffle formation (Figures 6G and 6H). Recruitment of MYO6 into ruffles at the plasma membrane depends entirely on the motor domain as constructs lacking the cargo-binding tail (Figure S7B) or with cargo-binding sites mutated (Figure S7C) was still observed in EGF-stimulated ruffles. Mutation of the motor domain at site T405, which has been shown to moderately affect duty ratio in vitro, did not affect recruitment of MYO6 into ruffles (Figure S7D).

In summary, our data indicate that MYO6 regulates peripheral actin structures through endosomal positioning and structural organization, thus providing a link between growth factor signaling and actin dynamics.

DISCUSSION

The actin cytoskeleton is emerging as a key regulator of the endocytic pathway. The varied architecture and dynamics of actin encountered by membranes at different steps in endosomal trafficking may determine the trajectory and rate of sorting and transport. In this study, we demonstrate that APPL1 endosomes, an early signaling compartment in the cell cortex that receives input directly from the plasma membrane, align along cortical actin filaments in the cell periphery, where they are anchored by the motor protein MYO6. Loss of MYO6 leads to accumulation of peripheral endosomes in the perinuclear space, dependent on microtubules. In contrast, reversing the direction of MYO6 leads to accumulation of APPL1 endosomes at the cell surface and clustering at the base of filopodia. Displacing endosomes toward the cell center leads to changes in both their biochemical composition and signaling function. AKT activation in response to EGF is significantly perturbed. Loss of MYO6 also impairs actin reorganization at the plasma membrane and membrane ruffle formation in response to growth factor stimulation, which may be linked to the reported role of signaling endosomes in RAC activation. These results indicate that spatial distribution is a key regulator of endosomal function. Our data can be summarized in the following model (Figure 7).

MYO6-Dependent Regulation of Endosomal Actin Architecture

Dynamic actin rearrangements are required throughout the endocytic pathway including for internalization at the plasma membrane, cargo sorting, membrane fission, and endosome distribution. Most published work has focused on actin rearrangements on early EEA1-positive sorting endosomes. These

Figure 6. MYO6 Is Required for Ruffle Formation

(A) HeLa cells were treated with APPL1 targeting siRNA, stimulated with EGF for 5 min, fixed, and stained with phalloidin. Yellow arrows (used throughout this panel) indicate the formation of membrane ruffles, which are not visible in cells lacking APPL1. Scale bar, 20 μm.

(B) HeLa cells were transfected with GFP-AKT-PH, treated with MYO6 siRNA, and stimulated with 100 ng/mL EGF for 5 min as in (A). Scale bar, 10 μm.

(C) Wild-type and MYO6-CRISPR-KO HeLa cells were stimulated with 20 ng/mL EGF for 5 min, fixed, and stained with anti-MYO6 antibody and phalloidin. Scale bar, 20 μm.

(D) Cells were transfected with YFP-RAC, fixed, and stained with phalloidin-Alexa 568 and mouse anti-RAB5 (detected with Alexa-647-labeled secondary anti-mouse antibodies).

(E) Expression of siRNA-resistant MYO6 rescues the defect in EGF-stimulated membrane ruffling. Cells were stimulated with 100 ng/mL EGF for 5 min, fixed, stained with phalloidin, and imaged in a wide-field microscope.

(F) Quantification of images in (E), with more than 300 cells per condition from two independent experiments.

(G) HeLa cells were transfected with the indicated GFP-tagged constructs, stimulated with 100 ng/mL EGF for 5 min, fixed, and stained with anti-GFP antibody and phalloidin. Scale bar, 20 μm.

(H) Quantification of experiments shown in (C), and (G), with more than 250 cells per condition from three independent experiments. Bar graphs represent mean ± SD.

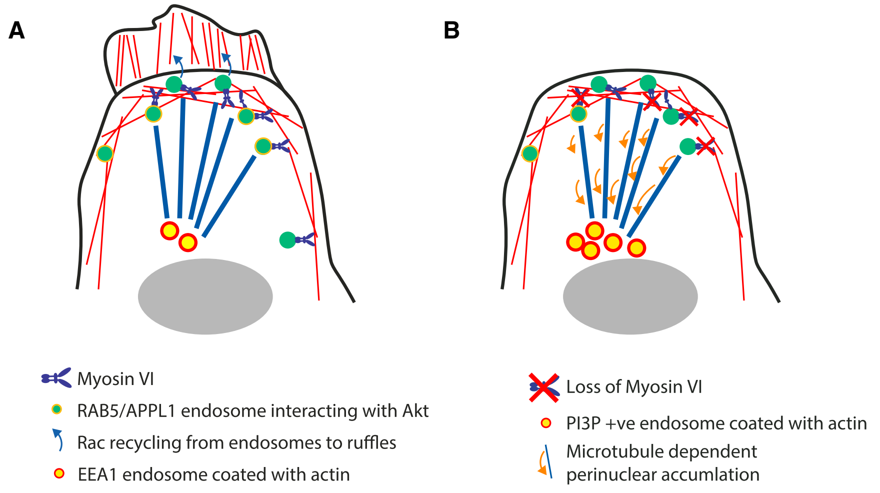

Figure 7. Model of the Role of MYO6 in Signaling Endosome Positioning and Function

(A) In wild-type cells, APPL1 endosomes sit on peripheral actin filaments. On stimulation with EGF, APPL1 recruits AKT promoting its activation. Retention of endosomes in the periphery, dependent on MYO6, further promotes AKT signaling. Formation of membrane ruffles may depend on RAC recycled from endosomes.

(B) When MYO6 is lost from APPL1 endosomes, they translocate to the perinuclear space where their lipid and protein composition is modified and F-actin accumulates. In this state, they cannot promote AKT activation or membrane ruffling in response to EGF.

EEA1 endosomes are coated by patches of actin, indicating these distinct steps of the endocytic pathway receive very different cues from the actin cytoskeleton, most likely via WASH-mediated polymerization (Derivery et al., 2009). Actin has been shown to regulate EEA1-positive endosome distribution, through ALIX (Cabezas et al., 2005) and Annexin A2 (Zobiack et al., 2003). By SIM, we show in this study that the arrangement of APPL1 and EEA1 endosomes with respect to actin is very different. Whereas EEA1 endosomes are often surrounded by an actin "cloud," the early signaling endosomes align along actin filaments like "beads on a string." The presence of MYO6 on APPL1 (but not EEA1) vesicles may relate to the character of actin to which they adhere, either by selectively binding to specific pre-existing filaments or regulating actin structure and polymerization once adhered. Selectivity for certain types of filaments may be driven by the presence of different isoforms of tropomyosin on different populations of actin (Gunning et al., 2015). Recent work indicates that actin-based motors may regulate their own actin tracks through a variety of mechanisms. For instance, MYO6 has been shown in a complex with the RACGEF DOCK7 (Majewski et al., 2012), while MYO9B contains a RHOGAP in its own tail domain (Wirth et al., 1996). Several studies have previously found a role for MYO6 in regulating actin organization and dynamics in a diverse array of biological functions including *Drosophila* spermatid individualization (Noguchi et al., 2006), melanosome biogenesis (Loubéry et al., 2012), and in cell-cell contacts (Mangold et al., 2011).

Endosome Distribution and Signaling Function

The spatial distribution of endosomes is closely linked to their function within intracellular signaling cascades. Following receptor internalization, nascent endosomes enter a unidirectional maturation pathway, which finally leads to the accumulation of early EEA1 endosomes, late endosomes, lysosomes, as well as recycling endosomes in the perinuclear space. Disruption of this pathway, e.g., caused by defects in MYO6-dependent endosome positioning, can lead to downstream effects, which may explain our previously observed reduction in membrane tubules emanating from the RAB11 recycling compartment (Chibalina et al., 2007). The close proximity of most endosomal compartments, except the APPL1 signaling endosomes, allows efficient cargo delivery between these different compartments and leads to the fast clearance of, for example, signaling receptors. A recent study has identified RNF26 as a crucial regulator of architecture in the endosomal system by orchestrating a ubiquitin-dependent vesicular tethering system in the perinuclear space (Jongsma et al., 2016). It is therefore crucial to actively exclude early signaling endosomes from the perinuclear space. The MYO6-dependent tethering of APPL1 endosomes to the actin cortex in the cell periphery stalls the endosomal maturation process and allows continued signaling, before downstream cargo processing. In this way MYO6 may act to oppose SQSTM1-mediated vesicular tethering to the endoplasmic reticulum (ER) (Jongsma et al., 2016), potentially constituting a cortical actin-localized counterbalancing component of a ubiquitin-mediated switch.

The Role of MYO6 and AKT in Cancer

Positioning of signaling endosomes mediated by MYO6 is crucial for their function. We showed that loss of MYO6 acutely perturbs phosphorylation of AKT on S473. We could find no defect in PI3K or AKT recruitment dynamics at the plasma membrane following EGF stimulation indicating that signaling endosome positioning is crucial for AKT phosphorylation. S473 is phosphorylated by TORC2 to promote cancer metastasis and invasion (Kim et al., 2011). Our observation that MYO6 plays a role in AKT signaling is an important finding that may have wider implications for the role of MYO6 in cancer cells, since this motor is dramatically overexpressed in prostate (Dunn et al., 2006) and ovarian (Yoshida et al., 2004) cancers. Furthermore, AKT activation has a crucial role in prostate cancer progression mainly driven by accumulation of plasma membrane PI(3,4,5)P_3 following mutations in PTEN (Majumder and Sellers, 2005). However, how AKT phosphorylation is coupled to the endosomal position is not clear, and the subcellular localization and activity of TORC2 remains mysterious. Reports suggest that TORC activity is promoted by RAC1 (Saci et al., 2011). Thus, an intimate feedback may exist between RAC1 recruitment to endosomes and

AKT phosphorylation. Displacement of signaling endosomes by knockdown of either MYO6 or APPL1 may thus affect both AKT activation and ruffle formation through RAC. EGFR can be trafficked through APPL1-positive endosomes to promote AKT activation (Scita and Di Fiore, 2010). However, depletion of MYO6 does not appear to affect EGFR uptake and degradation (Tumbarello et al., 2012), and the majority of EGFR may pass through a parallel pathway to APPL1 en route to EEA1 endosomes (Flores-Rodriguez et al., 2015). Thus, proximity of APPL1 endosomes to the plasma membrane and actin cortex may be required for their function in addition to their content. Many questions remain regarding the role of APPL1 in AKT activation.

MYO6 in Membrane Protrusion Formation

RAB5 endosomes have previously been shown to mediate activation of RAC, thereby regulating actin dynamics at the plasma membrane (Palamidessi et al., 2008). MYO6 is required for ruffle formation at the plasma membrane, suggesting that control of endosome position by MYO6 is a crucial step in the spatiotemporal regulation of RAC activity and thereby plasma membrane dynamics. However, MYO6 not only regulates plasma membrane protrusion formation, but is also present in membrane ruffles (Figures 6 and S7), where it has been suggested to mediate the polarized delivery of membrane into the leading edge by ensuring fusion of vesicles at the site of ruffle formation (Bond et al., 2011; Chibalina et al., 2010). Finally, at present we cannot exclude that this motor plays an additional direct mechanistic role in formation of plasma membrane ruffles by providing extra protrusion force when anchored to the plasma membrane and moving toward the minus end of actin filaments. In summary, the association of MYO6 with membrane ruffles and with endosomes required for RAC activation as well as the secretory pathway suggests that this motor may play a central role in coordinating the formation of protrusions at the plasma membrane with cellular signaling pathways.

EXPERIMENTAL PROCEDURES

Cell Lines, Antibodies, and Reagents

MEFs were derived from wild-type and Snells Waltzer (MYO6 knockout) mice as described previously (Warner et al., 2003) and cultured in DMEM supplemented with 2 mM glutamine, 10% fetal bovine serum, and penicillin/streptomycin. A549 cells were cultured in DMEM supplemented with Glutamax, 10% fetal bovine serum, and penicillin/streptomycin at standard concentration. HeLa cells were cultured in RPMI supplemented with high glucose, 10% fetal bovine serum (FBS), and penicillin/streptomycin. CRISPR knockout HeLa cells were generated as described (Brooks et al., 2017). siRNAs targeting MYO6 (smartpool and Oligo 7, J-0006355-07, sequence CAUUGUAUCUGGAG AAUCA), APPL1 (smartpool), TOM1 (smartpool), and N-WASP (smartpool) were obtained from Dharmacon (GE Healthcare). HeLa cells stably expressing GFP-MYO6-siRes, which is resistant to MYO6-targeting Oligo 7, was described previously (Tumbarello et al., 2012). PMT-RFP was a gift from Thorsten Wohlund (Liu et al., 2007). Lyn-mCherry (Plasmid 38004, deposited by Robin Irvine [Hammond et al., 2012]), GFP-AKT (Plasmid 39531 deposited by Julian Downward [Watton and Downward, 1999]), GFP-AKT-PH (Plasmid 21218 deposited by Tobias Meyer [Raucher et al., 2000]), and GFP-p85 (Plasmid 11499 deposited by Ronald Kahn was cloned into pEGFP-C3) were obtained from Addgene. GFP-MYO6 refers to the No Insert isoform (UniProtKB Q9UM54-5), and all amino acid designations align with this sequence. GFP-MYO6 mutants were as follows: GFP-MYO6-tail (Chibalina et al., 2010), GFP-MYO6-ΔPIP2 (Spudich et al., 2007); GFP-MYO6-K157R, GFP-MYO6-WLY, GFP-MYO6-RAL (Arden et al., 2016); GFP-MYO6-ΔTail, GFP-MYO6-T405A and GFP-MYO6-T405E (Brooks et al., 2017). GFP-MYO6-tail-RAL was generated by site-directed mutagenesis as described (Arden et al., 2016). Antibodies were anti-RAB5 (mouse monoclonal 610282, BD Biosciences), anti-GFP monoclonal (ab1218, Abcam), anti-GFP polyclonal (Invitrogen), Phalloidin-Alexa 568/647 (Invitrogen), anti-APPL1 (rabbit polyclonal, Santa Cruz Biotechnology), anti-EEA1 (mouse monoclonal 610456, BD Biosciences), anti-AKT (polyclonal, Cell Signaling Technology), anti-AKT-T308 (polyclonal, Cell Signaling Technology), anti-AKT-S473 (polyclonal, Cell Signaling Technology), anti-EGFR (polyclonal, Santa Cruz), MYO6 Ab2422 (developed in-house), MYO6 Ab3943 (developed in-house), MYO6 Ab9907 (developed in-house), anti-TOM1/TOM1L2 (ab96320, Abcam), and anti-N-WASP (polyclonal, Santa Cruz). All other reagents were from Sigma-Aldrich (Spudich et al., 2007)

Western Blotting

Cells at 70%–80% confluency were rinsed once in ice-cold PBS and lysed in ice-cold buffer containing 1% NP-40, 150 mM NaCl, 50 mM Tris, 1 mM EDTA, phosphatase inhibitor cocktail (Roche, Sigma), and protease inhibitor cocktail (Roche) for 5 min with scraping. Lysates were centrifuged at 20,000 × *g* (4°C) for 15 min, with the supernatant retained and either loaded on to 8% polyacrylamide gels (fabricated using the Bio-Rad system) of frozen at −80°C for later use. A two-sample t test was used to determine significance of experimental data averaged over three experiments. Blots were quantified using ImageJ. Total intensities for each band were extracted, background was subtracted, and phosphorylation data were scaled by the total amount of AKT in each lane. Multiple experiments were averaged by taking the phosphorylation signal at the 5-min time point as a constant, with changes measured relative to this time (set at 100%).

Immunofluorescence Sample Preparation

Cells were grown on no. 1.5 glass coverslips and following drug pre-treatment and/or EGF stimulation were rinsed once in room temperature Dulbecco's PBS and fixed in 4% formaldehyde in Dulbecco's PBS for 15 min. On some occasions a brief treatment (30 s) with 0.02% saponin was used prior to fixation to remove cytosolic proteins. Fixed cells were washed in PBS, permeabilized with 0.2% Triton in PBS for 5 min, washed again in PBS, and blocked in 1% BSA for 1 hr. Primary antibodies were added for 1 hr, followed by three washes in PBS for 5 min each. Coverslips were incubated with the appropriate Alexa-Fluor-labeled secondary antibodies (Invitrogen) for 1 hr, followed by a further three washes in PBS, rinsing in water, and mounting onto glass slides using Prolong Gold (Invitrogen). For SIM, cells were grown on acid-washed, no. 1.5, 18-mm square coverslips (high performance 170 ± 5 μm, Schott, Germany). Following permeabilization with Triton, cells were quenched twice with 0.2 M glycine for 5 min. Phalloidin was used at eight times the concentration of confocal experiments. Coverslips were mounted in Prolong Gold and cured for 3 days at room temperature in the dark prior to imaging.

Microscopy

Wide-field microscopy was performed using an AxioImager 2 (Zeiss) employing either a 63× or 100× objective. Confocal microscopy was performed with an LSM 880 (Zeiss). SIM was performed using an Elyra superresolution microscopy (Zeiss), using five rotations of the illumination pattern. Image processing (reconstruction and channel alignment) was conducted using Zen Black edition (Zeiss), and 3D rendering was performed using Volocity (Perkin-Elmer).

TIRF Assay

Cells were transfected with GFP-AKT-PH and a membrane marker control, either PMT-RFP or Lyn-mCherry. Cells were cultured in 35-mm glass-bottom coverslip dishes (81158, ibidi) and transfected with the relevant constructs using Fugene (Promega). Media was replaced with live-cell imaging solution (HEPES buffered, Invitrogen) and mounted on a TIRF microscope (Zeiss). Cells were illuminated through a 100× objective. Images were acquired every 10 s. Intensities were recorded for 2 min prior to EGF addition. As a control, media was added without EGF. To account for fluctuations in the amount of membrane in the TIRF field, GFP-AKT-PH intensity was divided by relative membrane marker intensity following background subtraction. The relative

recruitment of AKT-PH or AKT to the membrane was determined by dividing by the average value prior to EGF addition:

$$Recruitment(t) = \frac{\left(\frac{Akt_{Int}(t) - Akt_{BG}(t)}{PMT_{Int}(t) - PMT_{BG}(t)}\right)}{\left(\frac{Akt_{Int}(0) - Akt_{BG}(0)}{PMT_{Int}(0) - PMT_{BG}(0)}\right)}$$

where BG denotes background (determined from an area of the image outside the cell body.

Image Analysis and Statistics

Image processing was performed using ImageJ. The proportion of cells with aberrant large perinuclear aggregates of RAB5 vesicles was scored manually in each condition (with appropriate blinding controls). On average, ten fields of view each containing 20 cells for each condition were used per experiment, with each experiment repeated three times. Pearson's correlation coefficient was determined using the Coloc2 plug-in (in ImageJ)—images were background subtracted, and each cell was analyzed separately using a region of interest (ROI). The enrichment of GFP-PX on endosomes was calculated as follows. RAB5 endosomes were segmented using the Threshold function in ImageJ (the brightest 1% of pixels). The enrichment of GFP-PX on RAB5 endosomes was calculated as the ratio of endosomal GFP-PX fluorescence (using an ROI corresponding to segmented RAB5 endosomes) to total GFP-PX fluorescence in the cytoplasm, divided by the endosomal intensity of RAB5 (to account for increases in endosome density). For each experiment, the data were scaled such that the average enrichment in the MOCK cells was 1 (in arbitrary units). Statistics were determined using two sample t tests (Origin, OriginLab).

SUPPLEMENTAL INFORMATION

Supplemental Information includes Supplemental Experimental Procedures and seven figures and can be found with this article online at http://dx.doi.org/10.1016/j.celrep.2017.05.048.

AUTHOR CONTRIBUTIONS

T.A.M., M.V.C., D.A.T., and F.B. designed experiments; T.A.M., M.C., and D.A.T. performed experiments and analyzed data; F.B. initiated and coordinated the study; T.A.M. and F.B. wrote the paper.

ACKNOWLEDGMENTS

This work was funded by a Wellcome Trust University Award (086743) and project grants from the BBSRC (BB/K001981/1) and MRC (MR/K000888/1), awarded to F.B. CIMR is supported by the Wellcome Trust with a strategic award (100140) and equipment grant (093026).

Received: December 16, 2016
Revised: April 5, 2017
Accepted: May 12, 2017
Published: June 6, 2017

REFERENCES

Arden, S.D., Tumbarello, D.A., Butt, T., Kendrick-Jones, J., and Buss, F. (2016). Loss of cargo binding in the human myosin VI deafness mutant (R1166X) leads to increased actin filament binding. Biochem. J. *473*, 3307–3319.

Bond, L.M., Peden, A.A., Kendrick-Jones, J., Sellers, J.R., and Buss, F. (2011). Myosin VI and its binding partner optineurin are involved in secretory vesicle fusion at the plasma membrane. Mol. Biol. Cell *22*, 54–65.

Boulant, S., Kural, C., Zeeh, J.C., Ubelmann, F., and Kirchhausen, T. (2011). Actin dynamics counteract membrane tension during clathrin-mediated endocytosis. Nat. Cell Biol. *13*, 1124–1131.

Brooks, A.B.E., Humphreys, D., Singh, V., Davidson, A.C., Arden, S.D., Buss, F., and Koronakis, V. (2017). MYO6 is targeted by Salmonella virulence effectors to trigger PI3-kinase signaling and pathogen invasion into host cells. Proc. Natl. Acad. Sci. USA *114*, 3915–3920.

Broussard, J.A., Lin, W.H., Majumdar, D., Anderson, B., Eason, B., Brown, C.M., and Webb, D.J. (2012). The endosomal adaptor protein APPL1 impairs the turnover of leading edge adhesions to regulate cell migration. Mol. Biol. Cell *23*, 1486–1499.

Buss, F., Kendrick-Jones, J., Lionne, C., Knight, A.E., Côté, G.P., and Paul Luzio, J. (1998). The localization of myosin VI at the golgi complex and leading edge of fibroblasts and its phosphorylation and recruitment into membrane ruffles of A431 cells after growth factor stimulation. J. Cell Biol. *143*, 1535–1545.

Buss, F., Arden, S.D., Lindsay, M., Luzio, J.P., and Kendrick-Jones, J. (2001). Myosin VI isoform localized to clathrin-coated vesicles with a role in clathrin-mediated endocytosis. EMBO J. *20*, 3676–3684.

Cabezas, A., Bache, K.G., Brech, A., and Stenmark, H. (2005). Alix regulates cortical actin and the spatial distribution of endosomes. J. Cell Sci. *118*, 2625–2635.

Calleja, V., Alcor, D., Laguerre, M., Park, J., Vojnovic, B., Hemmings, B.A., Downward, J., Parker, P.J., and Larijani, B. (2007). Intramolecular and intermolecular interactions of protein kinase B define its activation in vivo. PLoS Biol. *5*, e95.

Chen, H.C., and Guan, J.L. (1994). Association of focal adhesion kinase with its potential substrate phosphatidylinositol 3-kinase. Proc. Natl. Acad. Sci. USA *91*, 10148–10152.

Cheng, J.Q., Lindsley, C.W., Cheng, G.Z., Yang, H., and Nicosia, S.V. (2005). The Akt/PKB pathway: Molecular target for cancer drug discovery. Oncogene *24*, 7482–7492.

Chibalina, M.V., Seaman, M.N.J., Miller, C.C., Kendrick-Jones, J., and Buss, F. (2007). Myosin VI and its interacting protein LMTK2 regulate tubule formation and transport to the endocytic recycling compartment. J. Cell Sci. *120*, 4278–4288.

Chibalina, M.V., Poliakov, A., Kendrick-Jones, J., and Buss, F. (2010). Myosin VI and optineurin are required for polarized EGFR delivery and directed migration. Traffic *11*, 1290–1303.

Derivery, E., Sousa, C., Gautier, J.J., Lombard, B., Loew, D., and Gautreau, A. (2009). The Arp2/3 activator WASH controls the fission of endosomes through a large multiprotein complex. Dev. Cell *17*, 712–723.

Driskell, O.J., Mironov, A., Allan, V.J., and Woodman, P.G. (2007). Dynein is required for receptor sorting and the morphogenesis of early endosomes. Nat. Cell Biol. *9*, 113–120.

Duleh, S.N., and Welch, M.D. (2010). WASH and the Arp2/3 complex regulate endosome shape and trafficking. Cytoskeleton *67*, 193–206.

Dunn, T.A., Chen, S., Faith, D.A., Hicks, J.L., Platz, E.A., Chen, Y., Ewing, C.M., Sauvageot, J., Isaacs, W.B., De Marzo, A.M., and Luo, J. (2006). A novel role of myosin VI in human prostate cancer. Am. J. Pathol. *169*, 1843–1854.

Eyster, C.A., Duggins, Q.S., and Olson, A.L. (2005). Expression of constitutively active Akt/protein kinase B signals GLUT4 translocation in the absence of an intact actin cytoskeleton. J. Biol. Chem. *280*, 17978–17985.

Flores-Rodriguez, N., Rogers, S.S., Kenwright, D.A., Waigh, T.A., Woodman, P.G., and Allan, V.J. (2011). Roles of dynein and dynactin in early endosome dynamics revealed using automated tracking and global analysis. PLoS ONE *6*, e24479.

Flores-Rodriguez, N., Kenwright, D.A., Chung, P.H., Harrison, A.W., Stefani, F., Waigh, T.A., Allan, V.J., and Woodman, P.G. (2015). ESCRT-0 marks an APPL1-independent transit route for EGFR between the cell surface and the EEA1-positive early endosome. J. Cell Sci. *128*, 755–767.

Gillham, H., Golding, M.C.H.M., Pepperkok, R., and Gullick, W.J. (1999). Intracellular movement of green fluorescent protein-tagged phosphatidylinositol 3-kinase in response to growth factor receptor signaling. J. Cell Biol. *146*, 869–880.

Gunning, P.W., Hardeman, E.C., Lappalainen, P., and Mulvihill, D.P. (2015). Tropomyosin - master regulator of actin filament function in the cytoskeleton. J. Cell Sci. *128*, 2965–2974.

Hammond, G.R.V., Fischer, M.J., Anderson, K.E., Holdich, J., Koteci, A., Balla, T., and Irvine, R.F. (2012). PI4P and PI(4,5)P2 are essential but independent lipid determinants of membrane identity. Science *337*, 727–730.

Heuser, J. (1989). Changes in lysosome shape and distribution correlated with changes in cytoplasmic pH. J. Cell Biol. *108*, 855–864.

Ikenoue, T., Inoki, K., Yang, Q., Zhou, X., and Guan, K.L. (2008). Essential function of TORC2 in PKC and Akt turn motif phosphorylation, maturation and signalling. EMBO J. *27*, 1919–1931.

Irie, H.Y., Pearline, R.V., Grueneberg, D., Hsia, M., Ravichandran, P., Kothari, N., Natesan, S., and Brugge, J.S. (2005). Distinct roles of Akt1 and Akt2 in regulating cell migration and epithelial-mesenchymal transition. J. Cell Biol. *171*, 1023–1034.

Jongsma, M.L., Berlin, I., Wijdeven, R.H., Janssen, L., Janssen, G.M., Garstka, M.A., Janssen, H., Mensink, M., van Veelen, P.A., Spaapen, R.M., and Neefjes, J. (2016). An ER-associated pathway defines endosomal architecture for controlled cargo transport. Cell *166*, 152–166.

Kim, D., Kim, S., Koh, H., Yoon, S.O., Chung, A.S., Cho, K.S., and Chung, J. (2001). Akt/PKB promotes cancer cell invasion via increased motility and metalloproteinase production. FASEB J. *15*, 1953–1962.

Kim, E.K., Yun, S.J., Ha, J.M., Kim, Y.W., Jin, I.H., Yun, J., Shin, H.K., Song, S.H., Kim, J.H., Lee, J.S., et al. (2011). Selective activation of Akt1 by mammalian target of rapamycin complex 2 regulates cancer cell migration, invasion, and metastasis. Oncogene *30*, 2954–2963.

Korolchuk, V.I., Saiki, S., Lichtenberg, M., Siddiqi, F.H., Roberts, E.A., Imarisio, S., Jahreiss, L., Sarkar, S., Futter, M., Menzies, F.M., et al. (2011). Lysosomal positioning coordinates cellular nutrient responses. Nat. Cell Biol. *13*, 453–460.

Lemmon, M.A., and Schlessinger, J. (2010). Cell signaling by receptor tyrosine kinases. Cell *141*, 1117–1134.

Lin, D.C., Quevedo, C., Brewer, N.E., Bell, A., Testa, J.R., Grimes, M.L., Miller, F.D., and Kaplan, D.R. (2006). APPL1 associates with TrkA and GIPC1 and is required for nerve growth factor-mediated signal transduction. Mol. Cell. Biol. *26*, 8928–8941.

Liu, P., Sudhaharan, T., Koh, R.M.L., Hwang, L.C., Ahmed, S., Maruyama, I.N., and Wohland, T. (2007). Investigation of the dimerization of proteins from the epidermal growth factor receptor family by single wavelength fluorescence cross-correlation spectroscopy. Biophys. J. *93*, 684–698.

Loubéry, S., Delevoye, C., Louvard, D., Raposo, G., and Coudrier, E. (2012). Myosin VI regulates actin dynamics and melanosome biogenesis. Traffic *13*, 665–680.

Majewski, Ł., Sobczak, M., Havrylov, S., Jóźwiak, J., and Rędowicz, M.J. (2012). Dock7: A GEF for Rho-family GTPases and a novel myosin VI-binding partner in neuronal PC12 cells. Biochem. Cell Biol. *90*, 565–574.

Majumder, P.K., and Sellers, W.R. (2005). Akt-regulated pathways in prostate cancer. Oncogene *24*, 7465–7474.

Mangold, S., Wu, S.K., Norwood, S.J., Collins, B.M., Hamilton, N.A., Thorn, P., and Yap, A.S. (2011). Hepatocyte growth factor acutely perturbs actin filament anchorage at the epithelial zonula adherens. Curr. Biol. *21*, 503–507.

Manning, B.D., and Cantley, L.C. (2007). AKT/PKB signaling: Navigating downstream. Cell *129*, 1261–1274.

Masters, T.A., and Buss, F. (2017). Filopodia formation and endosome clustering induced by mutant plus-end-directed myosin VI. Proc. Natl. Acad. Sci. USA *114*, 1595–1600.

Mitsuuchi, Y., Johnson, S.W., Sonoda, G., Tanno, S., Golemis, E.A., and Testa, J.R. (1999). Identification of a chromosome 3p14.3-21.1 gene, APPL, encoding an adaptor molecule that interacts with the oncoprotein-serine/threonine kinase AKT2. Oncogene *18*, 4891–4898.

Noguchi, T., Lenartowska, M., and Miller, K.G. (2006). Myosin VI stabilizes an actin network during Drosophila spermatid individualization. Mol. Biol. Cell *17*, 2559–2571.

Palamidessi, A., Frittoli, E., Garré, M., Faretta, M., Mione, M., Testa, I., Diaspro, A., Lanzetti, L., Scita, G., and Di Fiore, P.P. (2008). Endocytic trafficking of Rac is required for the spatial restriction of signaling in cell migration. Cell *134*, 135–147.

Park, H., Li, A., Chen, L.Q., Houdusse, A., Selvin, P.R., and Sweeney, H.L. (2007). The unique insert at the end of the myosin VI motor is the sole determinant of directionality. Proc. Natl. Acad. Sci. USA *104*, 778–783.

Petrie, R.J., Doyle, A.D., and Yamada, K.M. (2009). Random versus directionally persistent cell migration. Nat. Rev. Mol. Cell Biol. *10*, 538–549.

Raucher, D., Stauffer, T., Chen, W., Shen, K., Guo, S., York, J.D., Sheetz, M.P., and Meyer, T. (2000). Phosphatidylinositol 4,5-bisphosphate functions as a second messenger that regulates cytoskeleton-plasma membrane adhesion. Cell *100*, 221–228.

Saci, A., Cantley, L.C., and Carpenter, C.L. (2011). Rac1 regulates the activity of mTORC1 and mTORC2 and controls cellular size. Mol. Cell *42*, 50–61.

Sadowski, L., Pilecka, I., and Miaczynska, M. (2009). Signaling from endosomes: Location makes a difference. Exp. Cell Res. *315*, 1601–1609.

Saito, T., Jones, C.C., Huang, S., Czech, M.P., and Pilch, P.F. (2007). The interaction of Akt with APPL1 is required for insulin-stimulated Glut4 translocation. J. Biol. Chem. *282*, 32280–32287.

Sarbassov, D.D., Guertin, D.A., Ali, S.M., and Sabatini, D.M. (2005). Phosphorylation and regulation of Akt/PKB by the rictor-mTOR complex. Science *307*, 1098–1101.

Schenck, A., Goto-Silva, L., Collinet, C., Rhinn, M., Giner, A., Habermann, B., Brand, M., and Zerial, M. (2008). The endosomal protein Appl1 mediates Akt substrate specificity and cell survival in vertebrate development. Cell *133*, 486–497.

Scita, G., and Di Fiore, P.P. (2010). The endocytic matrix. Nature *463*, 464–473.

Spudich, G., Chibalina, M.V., Au, J.S.Y., Arden, S.D., Buss, F., and Kendrick-Jones, J. (2007). Myosin VI targeting to clathrin-coated structures and dimerization is mediated by binding to Disabled-2 and PtdIns(4,5)P2. Nat. Cell Biol. *9*, 176–183.

Stabley, D., Retterer, S., Marshall, S., and Salaita, K. (2013). Manipulating the lateral diffusion of surface-anchored EGF demonstrates that receptor clustering modulates phosphorylation levels. Integr. Biol. *5*, 659–668.

Tumbarello, D.A., Waxse, B.J., Arden, S.D., Bright, N.A., Kendrick-Jones, J., and Buss, F. (2012). Autophagy receptors link myosin VI to autophagosomes to mediate Tom1-dependent autophagosome maturation and fusion with the lysosome. Nat. Cell Biol. *14*, 1024–1035.

Varsano, T., Dong, M.Q., Niesman, I., Gacula, H., Lou, X., Ma, T., Testa, J.R., Yates, J.R., 3rd, and Farquhar, M.G. (2006). GIPC is recruited by APPL to peripheral TrkA endosomes and regulates TrkA trafficking and signaling. Mol. Cell. Biol. *26*, 8942–8952.

Varsano, T., Taupin, V., Guo, L., Baterina, O.Y., Jr., and Farquhar, M.G. (2012). The PDZ protein GIPC regulates trafficking of the LPA1 receptor from APPL signaling endosomes and attenuates the cell's response to LPA. PLoS ONE *7*, e49227.

Wang, Y., Pennock, S., Chen, X., and Wang, Z. (2002). Endosomal signaling of epidermal growth factor receptor stimulates signal transduction pathways leading to cell survival. Mol. Cell. Biol. *22*, 7279–7290.

Warner, C.L., Stewart, A., Luzio, J.P., Steel, K.P., Libby, R.T., Kendrick-Jones, J., and Buss, F. (2003). Loss of myosin VI reduces secretion and the size of the Golgi in fibroblasts from Snell's waltzer mice. EMBO J. *22*, 569–579.

Watton, S.J., and Downward, J. (1999). Akt/PKB localisation and 3′ phosphoinositide generation at sites of epithelial cell-matrix and cell-cell interaction. Curr. Biol. *9*, 433–436.

Wells, A.L., Lin, A.W., Chen, L.Q., Safer, D., Cain, S.M., Hasson, T., Carragher, B.O., Milligan, R.A., and Sweeney, H.L. (1999). Myosin VI is an actin-based motor that moves backwards. Nature *401*, 505–508.

Wirth, J.A., Jensen, K.A., Post, P.L., Bement, W.M., and Mooseker, M.S. (1996). Human myosin-IXb, an unconventional myosin with a chimerin-like rho/rac GTPase-activating protein domain in its tail. J. Cell Sci. *109*, 653–661.

Yoshida, H., Cheng, W., Hung, J., Montell, D., Geisbrecht, E., Rosen, D., Liu, J., and Naora, H. (2004). Lessons from border cell migration in the Drosophila ovary: A role for myosin VI in dissemination of human ovarian cancer. Proc. Natl. Acad. Sci. USA *101*, 8144–8149.

Zobiack, N., Rescher, U., Ludwig, C., Zeuschner, D., and Gerke, V. (2003). The annexin 2/S100A10 complex controls the distribution of transferrin receptor-containing recycling endosomes. Mol. Biol. Cell *14*, 4896–4908.

Zoncu, R., Perera, R.M., Balkin, D.M., Pirruccello, M., Toomre, D., and De Camilli, P. (2009). A phosphoinositide switch controls the maturation and signaling properties of APPL endosomes. Cell *136*, 1110–1121.

Cell Reports, Volume *19*

Supplemental Information

MYO6 Regulates Spatial Organization of Signaling Endosomes Driving AKT Activation and Actin Dynamics

Thomas A. Masters, David A. Tumbarello, Margarita V. Chibalina, and Folma Buss

Supplemental Information

Supplemental Experimental Procedures

Cloning of GFP-MYO6+. The 6 IQ domain of MYO5 was inserted into GFP-MYO6-NI (No Insert) by successive rounds of overlap extension PCR. The MYO6 tail was amplified from amino acid 913 to the C-terminal end with 5’GCGTGAGCTGAAGAAACTCAAAttacagaaaaaaaaacagcagg-3’ (the capitalised 5’ end of this oligo corresponds to the end of the 6IQ region of MYO5; amino acid 909) and 5’-TTTgcggccgcTTATTTCAACAGGTTCTGCAGC-3’ (containing a NotI site). The MYO5 motor domain and lever arm (amino acids 1-909) was amplified with oligos 5’-aaagaattctgatggctgcgtcggagctctacaca-3’ and 5’-cctgctgtttttttttctgtaaTTTGAGTTTCTTCAGCTCACGC-3’ (with the capitalised section corresponding to the reverse complement of the 3’ MYO5 sequence). These two products were then combined via an overlap extension reaction.

To create MYO6+, The MYO5 motor domain in this hybrid construct was replaced by the MYO6 motor domain by a similar approach. The motor domain of MYO6 (amino acids 1-770) was amplified by oligos 5’-caagaattcaaatggaggatggaaagccc-3’ and 5’-GGCAGCCCGAAGTTTGTCAGCcatgatctgatcaaattctgc -3’, where the capitalised region corresponds to the reverse complement of the 5’- end of the MYO5 lever arm. The MYO5 lever arm and MYO6-tail fusion were amplified by oligos 5’- gcagaatttgatcagatcatgGCTGACAAACTTCGGGCTGCC-3’ and 5’-TTTgcggccgcTTATTTCAACAGGTTCTGCAGC-3’ (as used in the first round). The two products were then fused by overlap extension to create the final construct; MYO6(1-770):MYO5(763-909):MYO6(913-end). This was cloned into pEGFP-C3 such that GFP was present at the N-terminus once expressed.

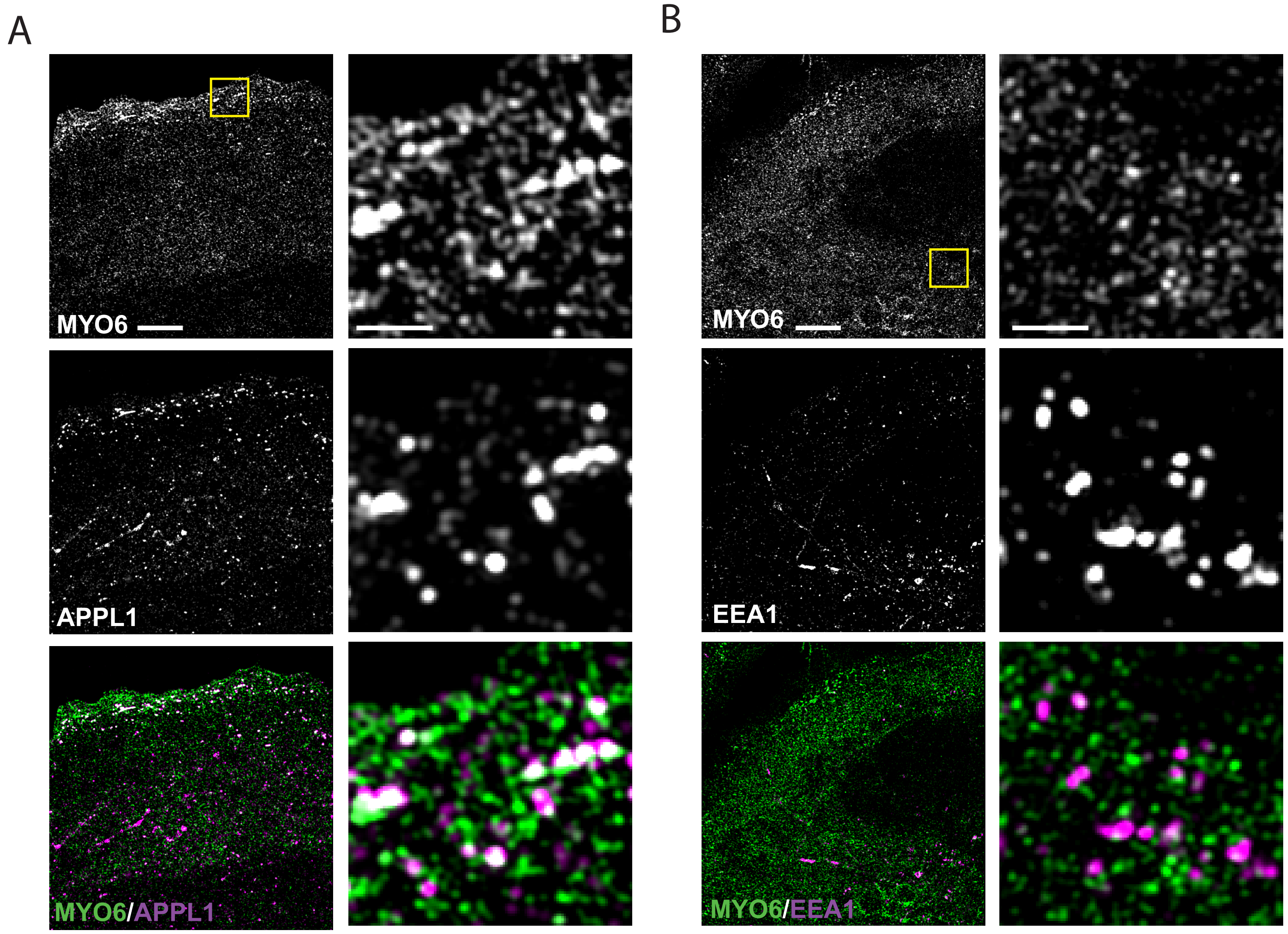

Figure S1, linked to Figure 1. MYO6 localises to APPL1 but not EEA1 endosomes. Hela cells were transfected with GFP-MYO6, fixed and stained with (A) anti-APPL1 polyclonal or (B) anti-EEA1 monoclonal antibodies, followed by imaging with Structured Illumination Microscopy. An overlay of MYO6 with APPL1 (MYO6/APPL1; green and magenta respectively) is presented in (A) together with the respective overlay with EEA1 in (B).

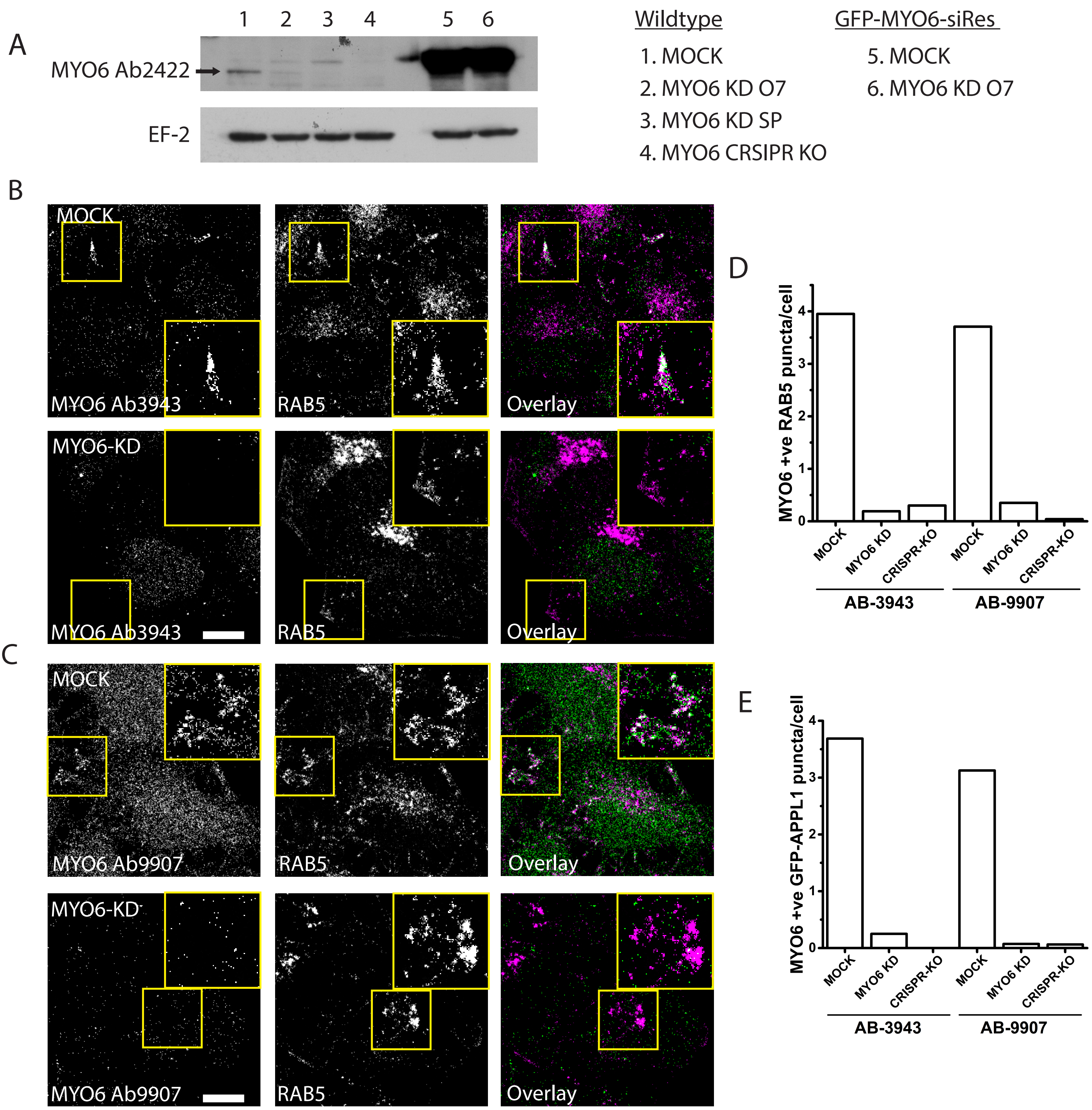

Figure S2, linked to Figure 2. Manipulation of endogenous MYO6 in Hela cells. (A) Western blotting of endogenous MYO6 (with homemade rabbit polyclonal anti-MYO6 antibody 2422) in wildtype and GFP-MYO6-siRes-expressing mock, siRNA Smartpool and O7 treated Hela cells. EF-2 is shown as loading control. (B) A homemade antibody to MYO6 (Ab3943) detects MYO6 on RAB5 puncta in mock but not MYO6 KD cells (scale bar 5 µm). (C) A further homemade antibody to MYO6 (Ab9907) detects MYO6 on RAB5 puncta in mock but not MYO6 KD cells (scale bar 5 µm). (D) Quantification of images in (A) and (B) and MYO6 positive-RAB5 puncta CRISPR MYO6 KO cells. (E) Quantification of GFP-APPL1 puncta positive for MYO6 in Hela MOCK, MYO6-KD and CRISPR MYO6 KO cells.

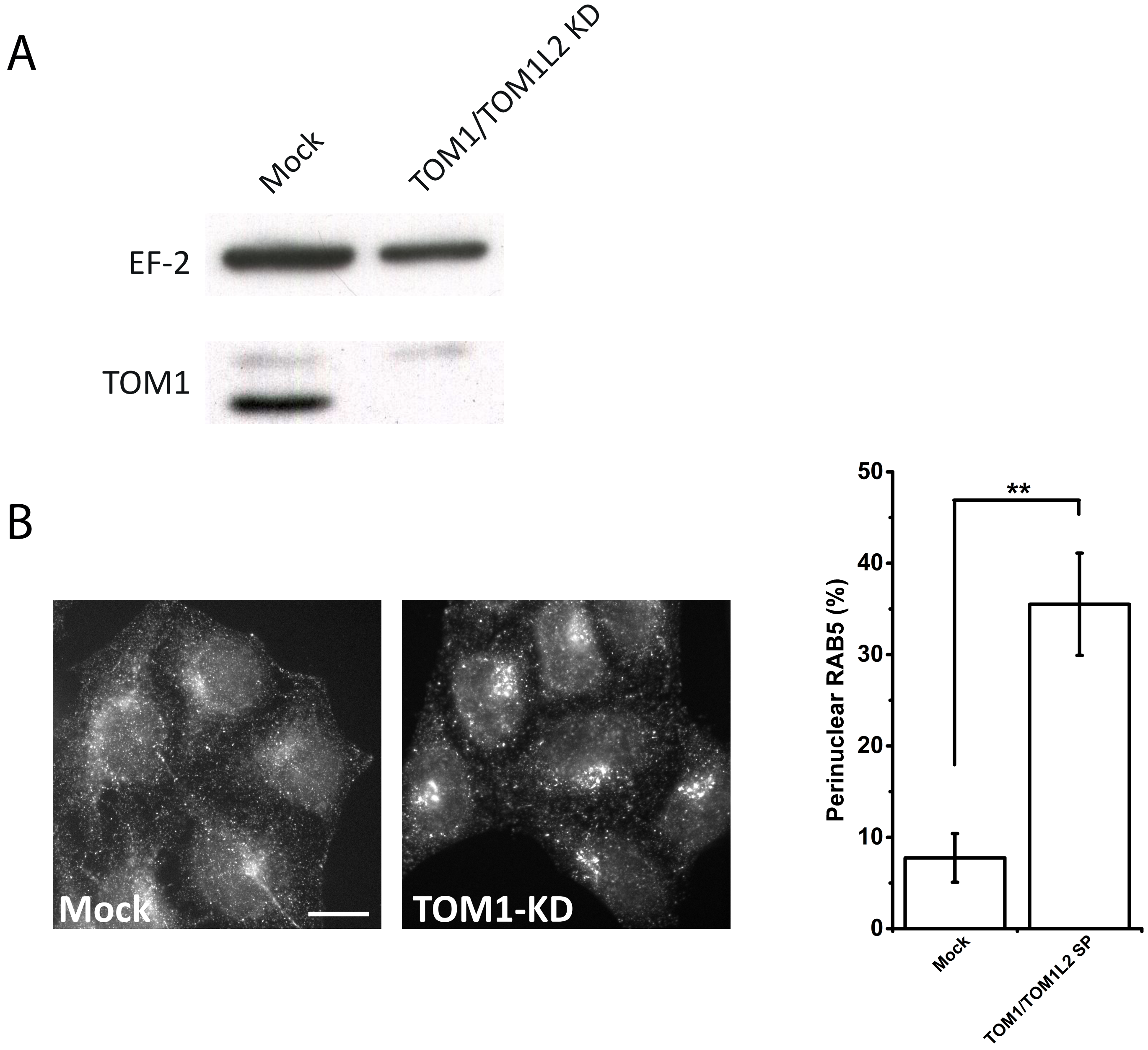

Figure S3, linked to Figure 2. Depletion of the endosomal MYO6 binding partner TOM1 leads to perinuclear accumulation of RAB5 endosomes. A) Cells were treated with Mock or siRNA targeting TOM1 and TOM1L2, lysed and subjected to Western blotting for TOM1 (EF-2 was used as a loading control). B) Cells were treated with Mock or siRNA targeting TOM1 and TOM1L2, fixed and stained with anti-RAB5 antibody. Cells with perinuclear accumulation of RAB5 were counted manually. Scale bar 10 µm. At least 300 cells per condition over 3 independent experiments were analysed. Bar graph represent mean ± S.D.

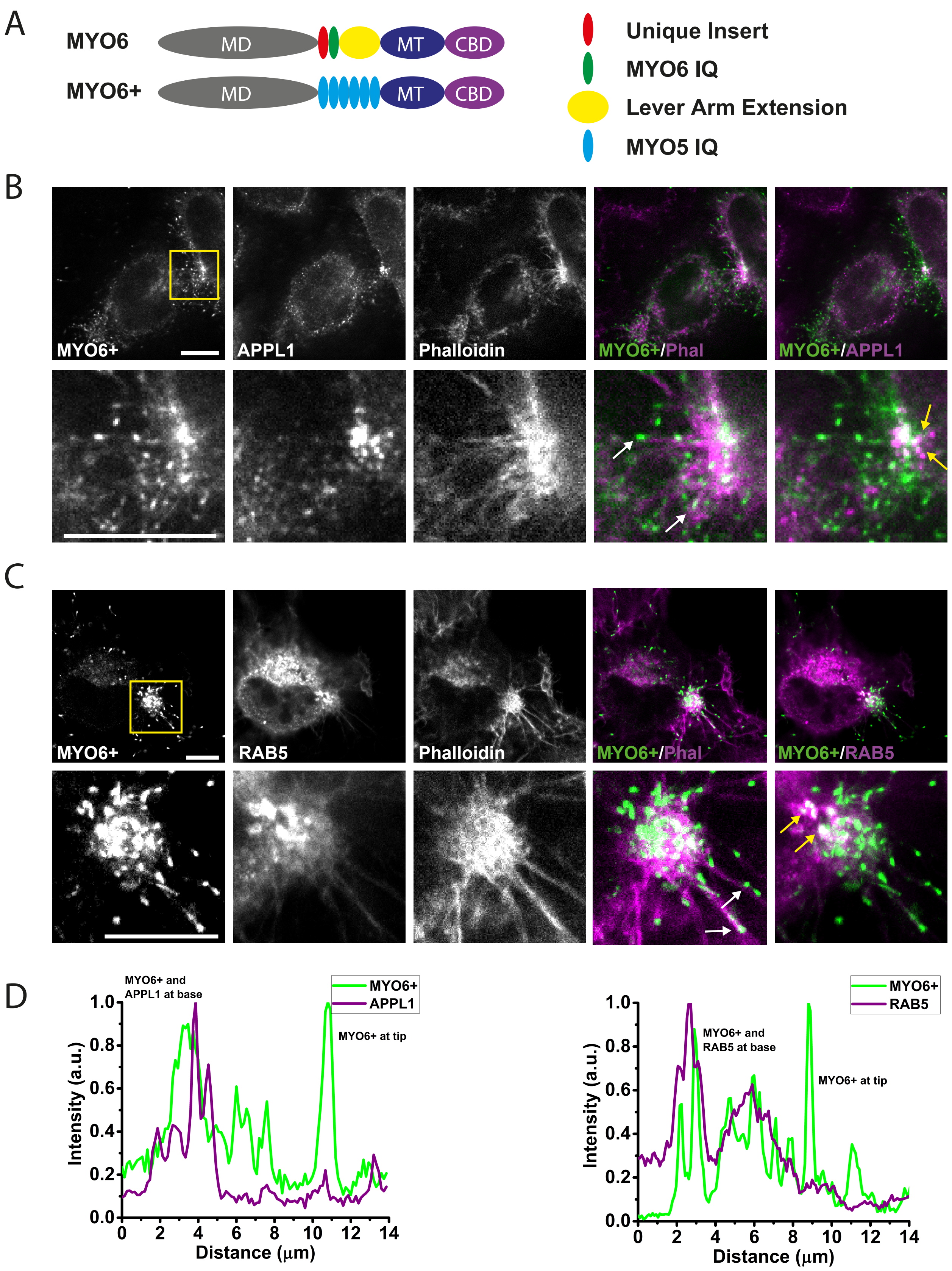

Figure S4, linked to Figure 2. A plus-end directed MYO6 mutant repositions APPL1- and RAB5-positive endosomes and re-organises cortical actin. (A) Schematic layout of MYO6+ and comparison to wild-type MYO6. (B). Hela cells were transfected with GFP-MYO6+, fixed and stained with anti-APPL1 polyclonal antibody and phalloidin. Overlays of mutant MYO6 with phalloidin (green and magenta respectively) and MYO6 with APPL1 (+/AP; green and magenta respectively) are presented. White arrows indicate filopodia tips and yellow arrows indicate APPL1 endosomes at the base. Scale bars 10 μm. (C). Hela cells were transfected with GFP-MYO6+, fixed and stained with anti-RAB5 monoclonal antibody and phalloidin. Overlays of mutant MYO6 with phalloidin (green and magenta respectively) and MYO6 with RAB5 (green and magenta respectively) are presented. White arrows indicate filopodia tips and yellow arrows indicate RAB5 endosomes at the base. Images are representative of three independent experiments. (D) Representative linescans of the images in (B) and (C) showing accumulation of RAB5 and APPL1 with MYO6+ at the base and an additional MYO6+ density at the tips of filopodia. Scale bars 10 μm.

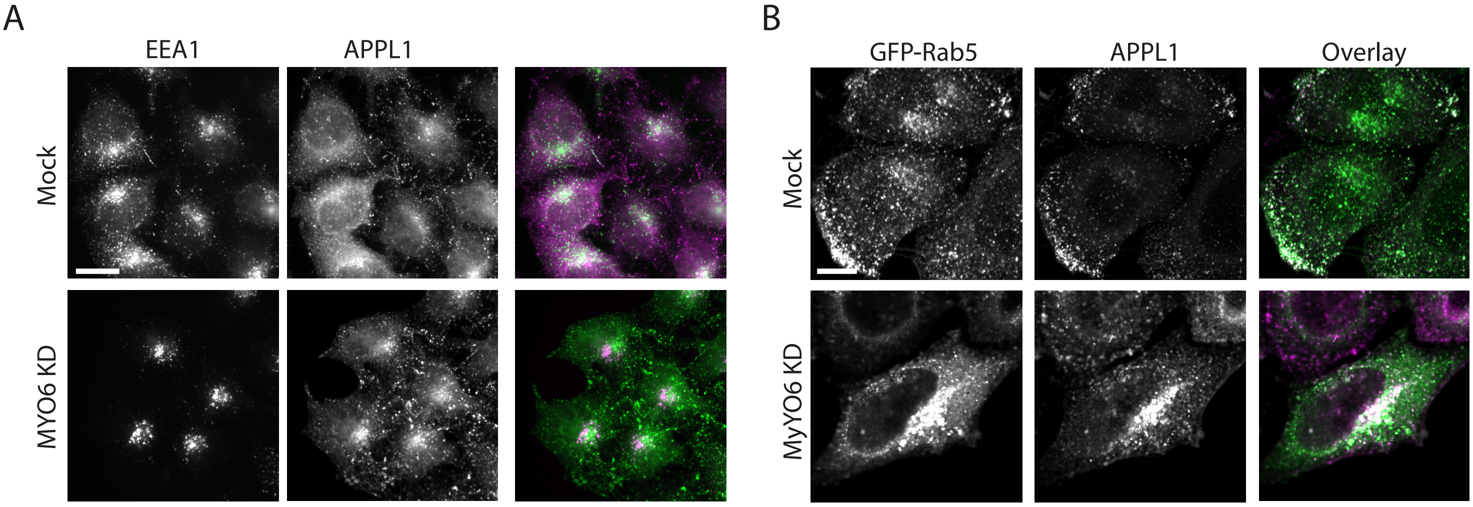

Figure S5, linked to Figure 3. Further characterisation of the endosomal system on acute depletion of MYO6. (A). Cells were treated with siRNA against MYO6, fixed and stained for EEA1 and APPL1. Scale bar 20 µm. (B). Cells were treated with siRNA against MYO6, transfected with GFP-RAB5, fixed and stained for APPL1. Scale bar 10 µm.

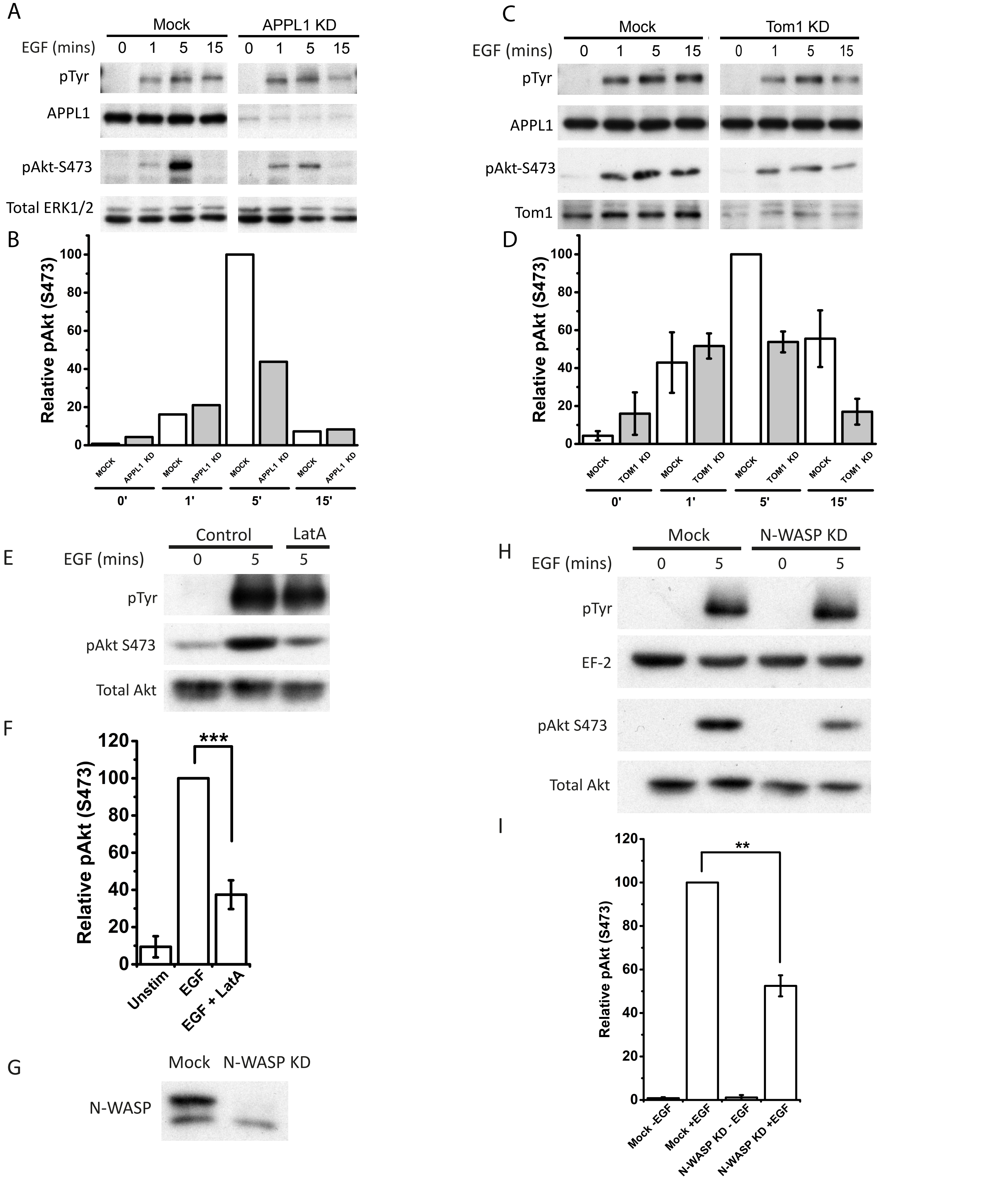

Figure S6, linked to Figure 4. The role of endosomes and actin networks in Akt phosphorylation. (A) A549 cells were treated with MYO6 or APPL1 siRNA and stimulated with 20 ng/ml EGF for the times indicated. Lysates were generated and blotted with the indicated antibodies. (B) Quantification of A. (C) A549 cells were treated with Tom1 siRNA and stimulated with 20 ng/ml EGF for 5 minutes. Lysates were generated and blotted with the indicated antibodies. (D) Quantification of (C) (3 independent experiments). (E) Cells were incubated with LatA, stimulated with EGF and lysates examined by western blot. (F) Quantification of (E) (6 independent experiments). (G) Cells were treated with N-WASP targeting siRNA and lysates were analysed by western blot. (H) Cells were treated with N-WASP targeting siRNA, stimulated with EGF and lysates were analysed by western blot. (I) Quantification of (H) (2 independent experiments). Bar graphs represent mean ± S.E.M.

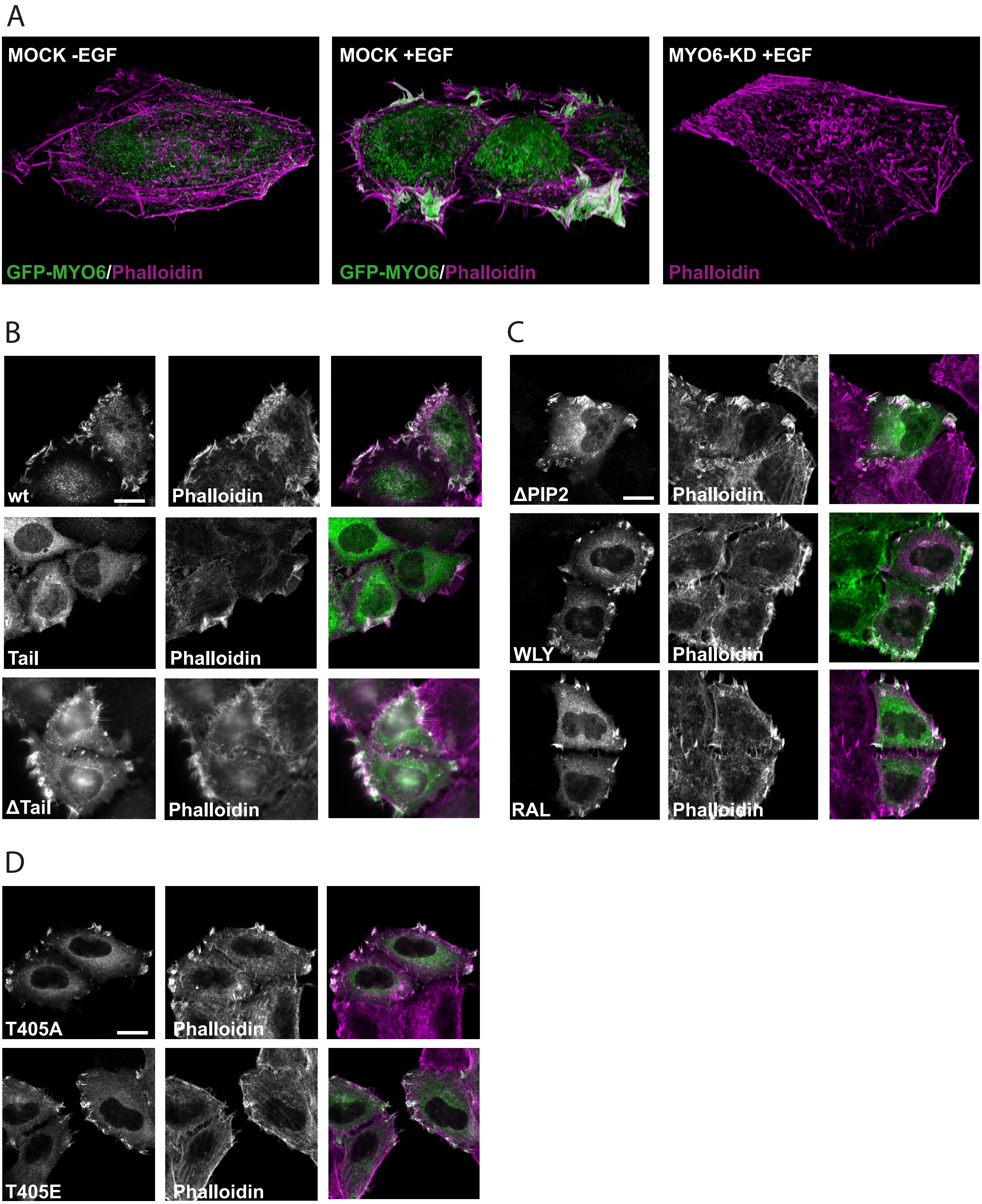

Figure S7, linked to Figure 6. MYO6 localises to ruffles through the head domain, independent of head phosphorylation state. Hela cells were transfected with the indicated MYO6 constructs, stimulated with 100 ng/ml EGF for 5 minutes and fixed. (A) Structured Illumination Microscopy shows both the structure of EGF-induced ruffles containing MYO6 (green), and F-actin structure (labelled by phalloidin, magenta) in mock unstimulated, mock EGF-stimulated and MYO6-siRNA-treated EGF-stimulated cells. (B) Wild-type GFP-MYO6, GFP-MYO6-Tail, GFP-MYO6-ΔTail. (C) GFP-MYO6-ΔPIP2, GFP-MYO6-WLY and GFP-MYO6-RAL. (D) GFP-MYO6-T405A and GFP-MYO6-T405E. Scale bars 20 µm.
